# Differential Analysis of Non-Volatile and Volatile Organic Compounds in *Lonicerae japonicae* Flos Across Four Geographical Origins of China Using HS-GC-IMS, HS-SPME-GC-MS, UPLC-Q-TOF-MS, and Multivariate Statistical Methods

**DOI:** 10.3390/molecules31010004

**Published:** 2025-12-19

**Authors:** Xiaobei Ning, Heng Lu, Lili Li, Minmin Zhang, Yujuan Jiang, Ibragimov Aziz Bakhtiyarovich, Xiao Wang, Iftikhar Ali, Wenhua Ji

**Affiliations:** 1 Key Laboratory for Applied Technology of Sophisticated Analytical Instruments of Shandong Province, Shandong Analysis and Test Center, Qilu University of Technology (Shandong Academy of Sciences), Jinan 250014, China; ningxbei@163.com (X.N.); locircle@163.com (H.L.); liliouc@126.com (L.L.); zhangminminff@163.com (M.Z.); wangx@sdas.org (X.W.); 2 Shandong C.P. Freda Pharmaceutical Co., Ltd., Jinan 250100, China; jyj0325@163.com; 3 Institute of General and Inorganic Chemistry of Uzbekistan Academy of Sciences, Mirzo Ulug’bek Str., 77a, Tashkent 100170, Uzbekistan; bobirjon_adizov@mail.ru; 4 Department of Chemistry, Karakoram International University, Gilgit 15100, Pakistan; lftikhar.ali@kiu.edu.pk

**Keywords:** solid-phase microextraction, ion mobility spectrometry, *Lonicerae japonicae* flos, chemometrics, liquid chromatography-mass spectrometry

## Abstract

Geographical origin constitutes one of the key factors that exert an influence on chemical compounds of *Lonicerae japonicae* flos (LJF). The present research was designed to explore differences among volatile organic compounds (VOCs) and non-VOCs among LJF samples from four geographical origins. Selection of 32 LJF samples with similar genetic backgrounds was performed using simple sequence repeat markers. Headspace solid-phase microextraction gas chromatography-mass spectrometry (HS-SPME-GC-MS) and headspace-gas chromatography-ion mobility spectrometry (HS-GC-IMS) were utilized to analyze VOCs, while non-VOCs were detected via ultra-high-performance liquid chromatography-quadrupole-time-of-flight mass spectrometry (UPLC-Q-TOF-MS). Multivariate statistical analyses were applied to screen differential compounds. The results indicated that HS-SPME-GC-MS and HS-GC-IMS identified 80 and 57 VOCs, respectively, with 34 key differential VOCs screened out, exhibiting significant variations among origins. For non-VOCs, 130 compounds were identified, with 19 key differential compounds showing geographical differences. This study further facilitates a comprehensive understanding of the chemical composition of LJF from different origins.

## 1. Introduction

*Lonicerae japonicae* flos (LJF), as the dried bud or flower initial blooming in *Lonicera japonica* Thunb. [1], has been widely used as both one of the most important traditional Chinese medicines (TCMs) and a dietary supplement or food throughout thousands of years in a Chinese context [2,3]. Bioactive components, encompassing not only numerous non-volatile organic compounds (non-VOCs, such as iridoids, phenolic acids, flavonoids, etc.) but also volatile organic compounds (VOCs, e.g., terpenoids, aldehydes, alcohols, and esters) [4] are plentiful in LJF. It exhibits diverse medicinal effects, including antibacterial, anti-inflammatory, and antioxidant properties [5,6]. The specific content and composition of these bioactive components can be affected by multiple factors, such as environmental factors, genetic traits, planting seasons, grower preferences, and storage conditions [7,8], with the environmental conditions of the origins being one of the critical factors [9]. Consequently, LJF originating from different geographical locations frequently demonstrates distinct chemical compositions. To explore this geographical specificity, the current study centers on LJF samples sourced from three major production provinces in China (Henan, Hebei, and Shandong), which are geographically adjacent yet feature distinct local environmental conditions. However, based on the Chinese Pharmacopeia, only four constituents, including 4,5-dicaffeoylquinic acid, cynaroside, chlorogenicacid, and 3,5-dicaffeoylquinic acid, are regarded as markers for quality evaluation [10]. However, the total content of three phenolic acids in LJF from different origins does not vary significantly [11], which limits research on differences in chemical components of LJF. Hence, it is crucial to develop a sophisticated and accurate approach to identify and analyze chemical components of LJF with different geographical origins.

Headspace solid-phase microextraction gas chromatography-mass spectrometry (HS-SPME-GC-MS) integrates the strengths of the high separation capacity of GC with the robust identification ability of MS [12,13]. It has been broadly used for quality classification and compound analysis of TCMs [14,15], especially for the detection of large molecular VOCs (carbon numbers ≥ 8). Headspace-gas chromatography-ion mobility spectrometry (HS-GC-IMS) combines the benefits of the high separation efficiency of GC with the fast response of IMS [16,17]. HS-GC-IMS has many advantages, including absence of a need for sample pretreatment, rapid detection, excellent resolution, intuitive data visualization, facility of operation, analytical speed and ultra-high sensitivity, and operation at atmospheric pressure [18,19]. More importantly, GC-IMS can detect small molecules (C_2_–C_10_) [20]. Therefore, the result of GC-IMS can be complementary to that of GC-MS, which provides a comprehensive analysis of VOCs of LJF.

As an important hyphenated method for characterizing the structure of components, ultra-high performance liquid chromatography (UHPLC) combined with quadrupole, hybrid orthogonal acceleration time-of-fight tandem mass spectrometry (Q-TOF-MS) has been increasingly used for the rapid identification of non-VOCs in TCMs due to its high resolution and unsurpassed sensitivity [21,22]. The components were characterized via comparison of retention times, accurate mass, MS fragmentation characteristic ions, and empirical formula with those reported for published compounds [23,24,25]. The combination of this method with HS-GC-IMS and HS-SPME-GC-MS have the capacity to exhaustively examine the chemical components of LJF.

Therefore, in this work, we selected LJF samples that have been cultivated extensively in the three major producing areas across China, aiming to detect VOCs and non-VOCs using HS-GC-IMS, HS-SPME-GC-MS, and UPLC-Q-TOF-MS. The key differential compounds in LJF were investigated using the variable importance projection (VIP) method based on the orthogonal partial least squares discrimination analysis (OPLS-DA) model. The findings provide a comprehensive approach to fully understanding the chemical compositions of LJF across different geographical origins, as well as their differential components.

## 2. Results and Discussion

### 2.1. Genetic Homogeneity of LJF

To assess genetic homogeneity of LJF, all the collected samples were subjected to molecular analysis using simple sequence repeat (SSR) markers. Samples were sent to Beijing Ruibo Xingke Biotechnology Co., Ltd., Beijing, China for SSR markers. Figure S1 was the dendrogram of the 62 LJF based on SSR markers. Thirty-two samples from Shandong, Henan, and Hebei with similar genetic backgrounds were selected for the experiment. The samples were divided into four groups: (a) Xinxiang (XX, 114.42° E, 35.05° N, *n* = 10); (b) Xingtai (XT, 115.04° E, 37.23° N, *n* = 8); (c) Heze (HZ, 115.95° E, 35.58° N, *n* = 5); and (d) Linyi (LY, 117.62° E, 35.52° N, *n* = 9). Table S1 provides a general overview of the environmental conditions for the four origins.

### 2.2. Analysis of VOCs

#### 2.2.1. Analysis of VOCs by HS-GC-IMS

**Figure 1:**
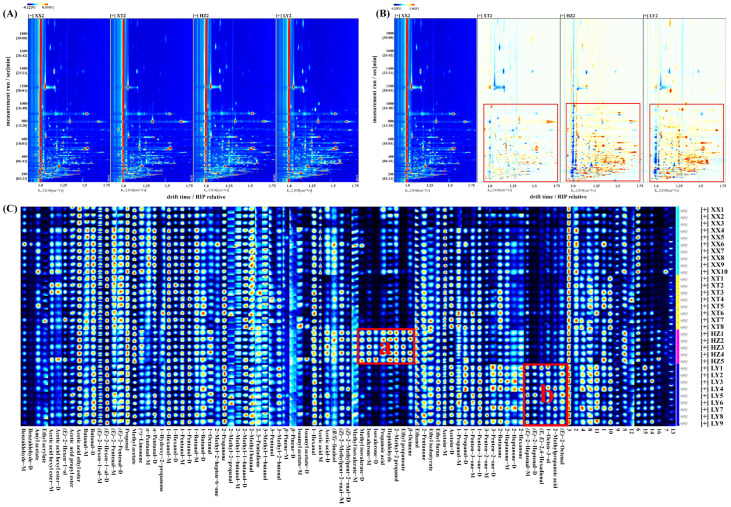
Comparison of VOCs of LJF from XX, XT, HZ, and LY regions detected by HS-GC-IMS. (**A**) Two-dimensional topographic plots; (**B**) comparative difference spectrum of two-dimensional topographic plots; (**C**) gallery plot. “M” and “D” denote monomer and dimer, respectively (1–16 numbers: unidentified compounds).

The VOCs in LJF from four origins were subjected to analysis via HS-GC-IMS. QC samples were analyzed (*n* = 9). Figure S2 illustrates the comparative difference spectrum of two-dimensional topographic plots. Peak volumes were calculated by relative standard deviation (RSD). For QC samples, 98.9% of VOCs had RSD values less than 30%, indicating good stability and repeatability of analysis. The two-dimensional spectrum (Figure 1A) shows the vertical axis representing retention time of GC and the horizontal axis representing drift time of IMS. The red vertical line denotes the reaction ion peak (RIP). Every signal point on the right side of RIP stands for one type of VOC. As a result of adducts forming between reaction ions and neutral molecules, a single VOC at high concentration might produce multiple signals (monomers or dimers) [26]. To enable clearer visualization of variations in the spectra of LJF samples from different origins, the spectra underwent normalization for comparative analysis. As shown in Figure 1B, white, blue, and red denote similar, lower, and higher levels of VOCs, respectively [27]. Over the range of retention time from 100 to 1000 s, lighter blue and red signals in XT groups, accompanied by more white signal dots, indicate comparable VOC content and types without notable variations despite differences between XX and XT groups. By contrast, a significant number of red dots in the HZ and LY groups indicates substantially higher VOCs content than in XX groups.

As listed in Table S2, 57 distinct VOCs (C_2_–C_10_) were tentatively identified, including 16 aldehydes, 12 ketones,11 alcohols, 10 esters, 4 terpenoids, 3 acids, and 1 furan. In addition, 16 peaks remained unidentified. From fingerprints, it could be visually seen that the differences in VOCs between samples. As shown in Figure 1C, the characteristic fingerprints are categorized into two regions. In region a, the content of methyl isovalerate, isovalerone, propanoic acid, heptaldehyde, 2-methyl-2-propanol, ethyl propanoate, and *β*-ocimene in HZ are higher. The five VOCs in region b had the higher content in LY, including (*E*)-2-heptenal, (*E*, *E*)-2,4-hexadienal, 1-octen-3-ol, 2-methylpropanoic acid, and (*E*)-2-octenal.

#### 2.2.2. Analysis of VOCs by HS-SPME-GC-MS

**Figure 2:**
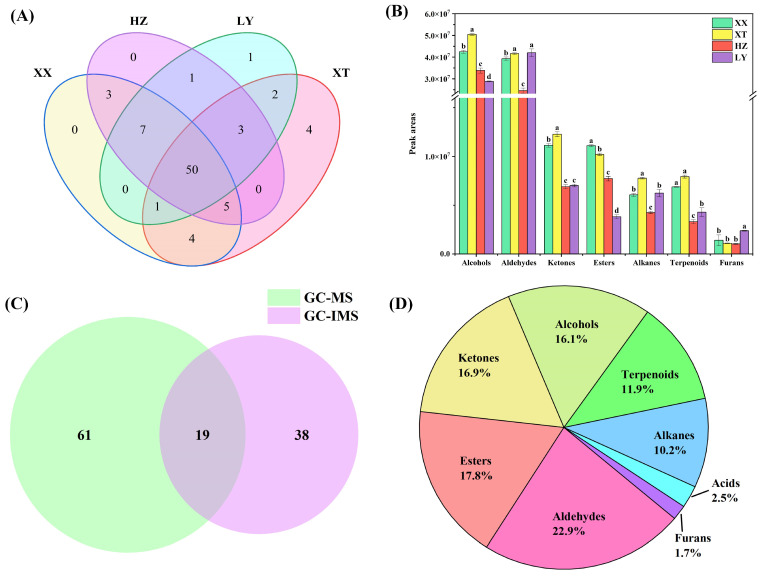
Comparison of VOCs of LJF from XX, XT, HZ, and LY origins. (**A**) Venn diagram (GC-MS); (**B**) peak area comparison of different origins of volatile compounds. Values with different letters are significantly different (*p* < 0.05) (GC-MS); (**C**) Venn diagram (GC-IMS and GC-MS); (**D**) proportion of different origins of volatile compounds (GC-IMS and GC-MS).

QC samples were analyzed (*n* = 9). Figure S3 illustrates the chromatogram. The overlap of retention time and peak intensity demonstrates acceptable system stability during the experimental process. Peak areas were calculated by RSD. For QC samples, 89.7% of VOCs had RSD values less than 30%. As listed in Table S3, a total of 80 VOCs, including 20 aldehydes, 14 alcohols, 12 esters, 12 alkenes, 11 terpenoids, 10 ketones, and 1 furan, were identified using HS-SPME-GC-MS. Among them, there are 63 compounds with carbon numbers ≥ C8. As shown in Figure 2A, 50 VOCs are identified across four different origins. Notably, certain VOCs are exclusively detected in specific origins. For example, *trans*-2-hexen-1-ol is only detected in LY samples. Spathulenol, *trans*-nerolidol, jasmone, and tridecane are only detected in XT samples. These exclusive occurrences may be linked to environmental differences: higher precipitation in LY could promote alcohols such as *trans*-2-hexen-1-ol [28], whereas the warmer, sunnier, and drier conditions in XT might favor terpenoids [29] like spathulenol, highlighting how climatic factors drive VOC divergence in LJF.

The distributions of VOCs across origins are illustrated in Figure 2B, with aldehydes and alcohols being the dominant components in terms of content. The analysis of significant differences is based on *p* < 0.05. Significant differences are observed across origins for aldehydes/ketones and ketones/alkenes in XX and XT, respectively, with each region compared to others. The significant differences compared with other origins for aldehydes/alkenes/terpenoids and furans/terpenoids in HZ and LY are also indicated, respectively. For alcohols and esters, significant differences between four origins are observed. Table S3 shows that the relative content of the top 10 VOCs by peak area exceeded 60% in each origin, making them major contributors. The top 10 VOCs share six core compounds, including benzaldehyde, 1-octanol, 1-hexanol, hexanal, 2-phenylethanol, and 3,5-octadien-2-one. Certain compounds also show higher abundance in the top 10 VOCs of specific origins: 3-octen-2-one in XT, methyl palmitate in HZ, and 2-hexenal/*trans*-2-hexenal in LY, reflecting geographical specificity in their abundance. These indicate geographical origin influences VOC compositions.

#### 2.2.3. Combined Analysis of VOCs by HS-GC-IMS and HS-SPME-GC-MS

57 VOCs are detected by HS-GC-IMS, while 80 VOCs are detected by HS-SPME-GC-MS. Among them, 19 VOCs are found to be common to both methods (Figure 2C), including 9 aldehydes (benzaldehyde, (*E*)-2-octenal, *trans*-2-heptenal, *trans*-2-hexenal, heptanal, (*E*)-2-pentenal, hexanal, pentanal, and octanal), 6 alcohols (1-octen-3-ol, *trans*-2-hexen-1-ol, 1-hexanol, 1-pentanol, 2-methyl-1-butanol, and isoamyl alcohol), 2 ketones (2-heptanone and 6-methyl-5-hepten-2-one), 1 terpenoids (linalool), and 1 ester (hexyl acetate). As illustrated in Figure 2D, the classification of 118 VOCs further shows that the combination of HS-GC-IMS and HS-SPME-GC-MS classify the detected VOCs into nine categories, including 27 aldehydes, 21 esters, 20 ketones, 19 alcohols, 12 alkenes, 14 terpenoids, 3 acids, and 2 furans. A comparative analysis with the previous literature on VOCs of LJF [30,31,32] indicated the discovery of several newly identified compounds, including *α*-terpineol, *trans*-nerolidol, *β*-cyclocitral, and *β*-ionone, which may potentially enrich the known volatile profile of LJF.

#### 2.2.4. Multivariate and Differential Analysis for VOCs

**Figure 3:**
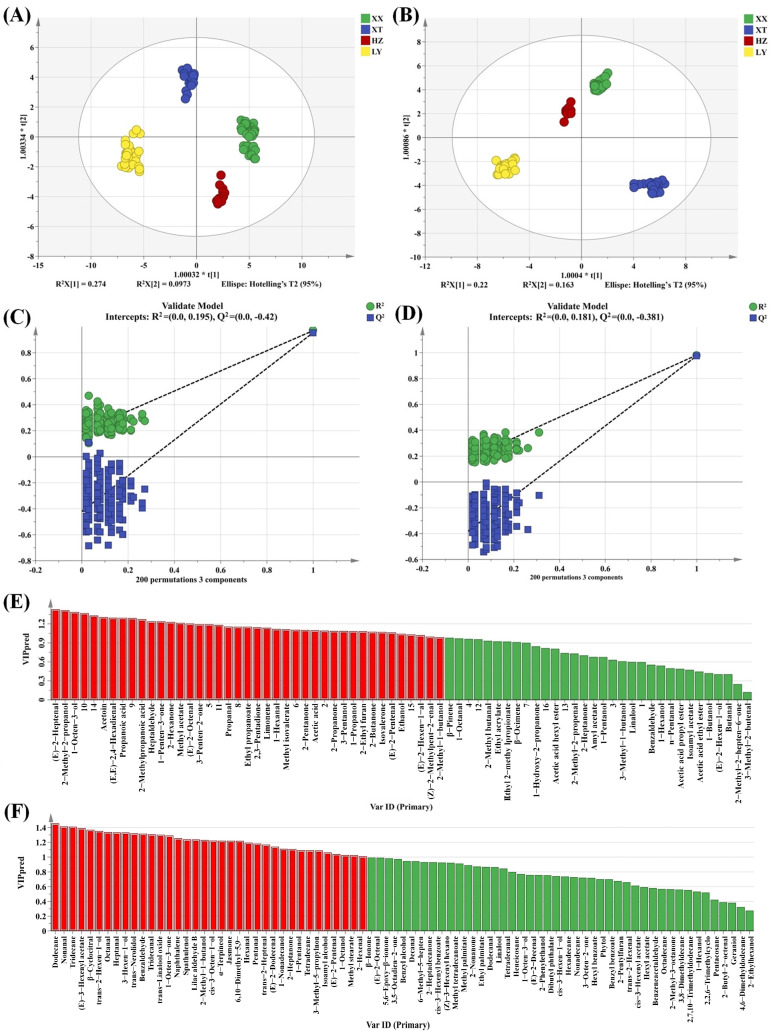
OPLS-DA score plots based on GC-IMS (**A**) and GC-MS (**B**); the 200 permutation tests based on GC-IMS (**C**) and GC-MS (**D**); VIP plot based on GC-IMS (**E**) and GC-MS (**F**) (the red part represents VIP > 1).

As a supervised discriminant analysis technique, OPLS-DA can maximize the separation between observation groups and has better predictive power and classification ability than PCA, incorporating grouping variables to compensate for the limitations of PCA [33]. Thus, the differences in LJF among four origins can be evaluated by OPLS-DA analysis. Two OPLS-DA models are established using HS-GC-IMS (Figure 3A) and HS-SPME-GC-MS (Figure 3B). Evidently, the two OPLS-DA score plots display significant inter-group differences in VOCs among samples from four origins. The VOCs characteristics of HZ and XX are relatively similar. The prediction parameters of the HS-GC-IMS OPLS-DA model (R^2^X = 0.875, R^2^Y = 0.954, and Q^2^ = 0.940) and the HS-SPME-GC-MS OPLS-DA model (R^2^X = 0.578, R^2^Y = 0.981, and Q^2^ = 0.972) show that both R^2^Y and Q^2^ scores are greater than 0.9, indicating that the two models have good predictive ability [34]. To prevent overfitting, 200 permutation tests are conducted to verify the fitness of the two OPLS-DA models. The R^2^ and Q^2^ values on the left are lower than those on the right, demonstrating that the two models are reliable and stable (Figure 3C,D). Variable importance in projection (VIP) is utilized to evaluate the explanatory ability and influence strength of each variable in discrimination and classification [35]. When the VIP value of a variable exceeded 1, it is regarded as playing an important role. As shown in Figure 3E,F, a total of 30 and 36 differential compounds with VIP > 1 are screened for HS-GC-IMS and HS-SPME-GC-MS, respectively.

**Figure 4:**
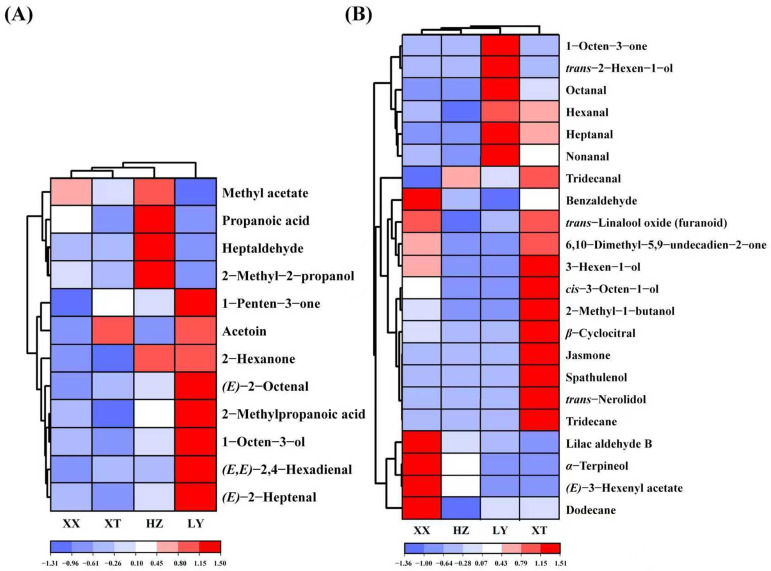
Heatmap of 12 VOCs by GC-IMS (**A**) and 22 VOCs by GC-MS (**B**).

Double variable criterion (VIP > 1.2 and *p* < 0.05) to screen key differential compounds. A total of 12 and 22 compounds are screened out by HS-GC-IMS and HS-SPME-GC-MS, respectively. Hierarchical clustering heatmap can visualize the distribution of differential compounds in different origins (Figure 4A,B). Based on these two methods, a total of 34 differential VOCs were screened out, and their VIP values are listed in Table S4. Overall, the 34 compounds can be categorized into seven groups (11 aldehydes, 6 alcohols, 4 ketones, 7 terpenoids, 2 esters, 2 acids, and 2 alkenes).

(*E*)-2-Octenal with the insect-repellent activity is most abundant in LY samples, which may enhance pest repellency [36]. (*E*)-2-Heptenal and 1-octen-3-ol can inhibit Aspergillus flavus and reduce the production of aflatoxin [37,38], which at higher levels in LY samples may reduce post-harvest contamination. Other VOCs can contribute to the aroma profile. For example, benzaldehyde, *α*-terpineol, and *trans*-linalool oxide are abundant in XX samples, which can enhance almond odor, floral, fruity, and woody aromas [39,40]. Spathulenol, *trans*-nerolidol, and jasmone are exclusive in XT samples, possibly caused by the unique environment. 6,10-Dimethyl-5,9-undecadien-2-one and *β*-cyclocitral are also higher content in XT samples. These compounds can enhance floral, fruity, and woody aromas. In addition, 3-hexen-1-ol in XT samples is most abundant which can contribute fresh herbal notes [41]. Table S1 indicates that XT exhibits a higher temperature, more sunshine, and lower precipitation compared to XX. These conditions may enhance the production of aromatic terpenoids and benzenoids [42,43], which contributes to the richer floral and fruity aromas observed in XT.

### 2.3. Analysis of Non-VOCs

#### 2.3.1. Analysis of Non-VOCs by UPLC-Q-TOF-MS

**Figure 5:**
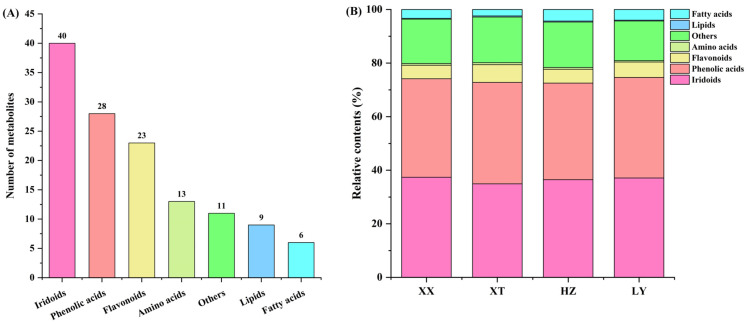
Comparison of non-VOCs of LJF from XX, XT, HZ and LY origins detected by UPLC-Q-TOF-MS. (**A**) The number of different categories; (**B**) the relative content of different categories.

QC samples were analyzed by UPLC-Q-TOF-MS (*n* = 9). Figure S4 illustrates the total ion current chromatograms of QC samples for positive ion mode (ESI+) and negative ion mode (ESI-). Retention time and peak intensity basically overlapped, indicating the weakness of perturbation during the test. Peak areas were calculated by RSD. For QC samples, 96.9% of non-VOCs had RSD values less than 30%, indicating good stability and repeatability of analysis. In LJF, a total of 130 non-VOCs were identified by comparison with the reported literature [44], comprising 40 iridoids, 28 phenolic acids, 23 flavonoids, 13 amino acids, 9 lipids, and 6 fatty acids, as well as 11 other types of compounds (Figure 5A); for details refer to Table S5. The relative contents of seven categories of non-VOCs were depicted in Figure 5B. The relative contents of iridoids and phenolic acids are in a dominant position in four origins samples. In four origins, the relative contents of iridoids and phenolic acids are comparable, but the number of phenolic acids is obviously lower than that in iridoids. Despite fewer types being detected for fatty acids compounds, compared to lipids and amino acids, the relative content of the former is higher than that of the latter among four origins.

#### 2.3.2. Multivariate and Differential Analysis for Non-VOCs

**Figure 6:**
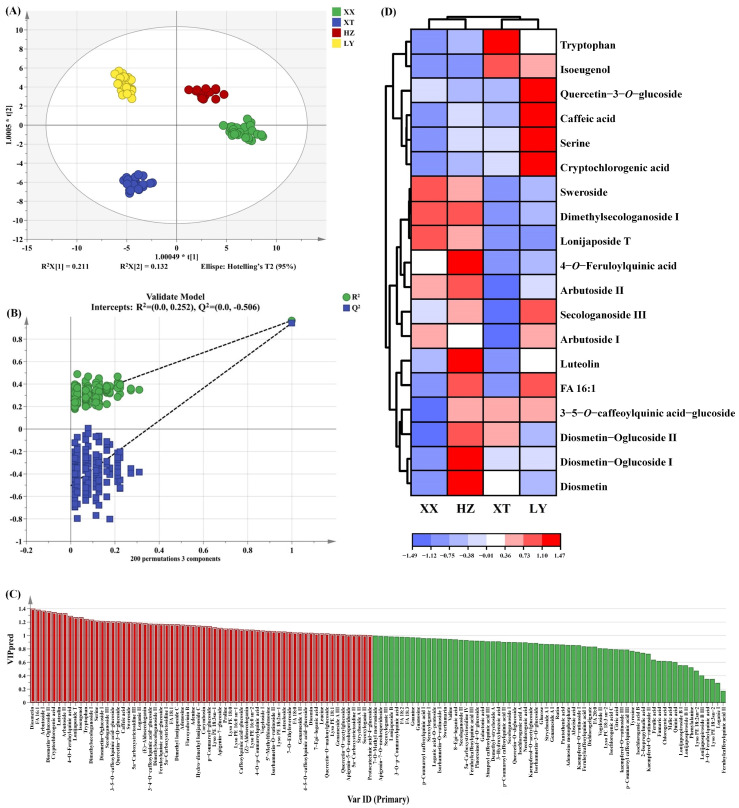
The OPLS-DA results of LJF from XX, XT, HZ, and LY origins by UPLC-Q-TOF-MS. (**A**) Score plots of OPLS-DA; (**B**) cross-validation plot by 200 permutation tests; (**C**) the red part represents 64 differential compounds with VIP > 1 (the red part represents VIP > 1); (**D**) hierarchical clustering heatmap of 19 key differential compounds (VIP > 1.2 and *p* < 0.05).

The OPLS-DA score plot is shown in Figure 6A, which clearly illustrates the distinct distribution of LJF samples. XX and HZ are positioned nearest, indicating that the non-VOCs are relatively similar, which is consistent with the results of VOCs analysis as mentioned above. The model of OPLS-DA yielded an R^2^X of 0.886, an R^2^Y of 0.965, and a Q^2^ of 0.941, indicating good predictive capability. The 200 permutation tests were performed to confirm that there was no overfitting in the OPLS-DA model (Figure 6B). As shown in Figure 6C, 64 non-VOCs differential compounds with VIP > 1 were screened out.

To identify key differential compounds, we further screened the substances based on double variable criterion (VIP > 1.2 and *p* < 0.05). Nineteen differential compounds were screened out, including 6 iridoid, 5 phenolic acids, 5 flavonoids, 2 amino acids, and 1 fatty acid. To visualize differential compound abundance across geographical origins of LJF, a hierarchical clustering heatmap analysis was performed on the 19 compounds (Figure 6D). The analysis reveals distinct differences among the LJF samples, highlighting their important role in distinguishing LJF samples. The VIP of the above 19 key non-VOCs are listed in Table S4.

The content of phenolic acid correlates positively with altitude within a range [45]. Cryptochlorogenic acid and caffeic acid in LY samples are more abundant, which may be due to more mountainous terrain and higher altitudes of LY. HZ samples have the highest level of luteolin, which can enhance anti-inflammatory and antibacterial activities [46]. XT samples show high contents of diosmetin which can exhibit anticancer effects [47]. Samples from XX have abundant sweroside, a compound which has activity in protecting nerves [48]. These findings collectively demonstrate that the chemical composition of LJF varies across different growing regions, and these variations are closely linked to local environmental and climatic factors, including temperature, precipitation, and topography.

## 3. Materials and Methods

### 3.1. Samples and Reagents

Sixty-two samples from Shandong, Henan, Hebei, Guizhou, and Yunnan in China were collected. Authentication of the LJF samples was conducted by Professor Xiao Wang at Qilu University of Technology. All LJF samples were dried in an oven at 40 °C, and the moisture content of all dried samples was below 10%. Each sample was pulverized to powder form.

The HPLC grades, including formic acid, acetonitrile, and methanol were obtainted form Merck (Darmstadt, Germany). *n*-Ketones (C_4_–C_9_) were bought from Sigma-Aldrich (St. Louis, MO, USA). A Direct-Q 8 UV-R water purification system (Millipore, Billerica, MA, USA) was used to generate ultrapure water.

### 3.2. HS-GC-IMS Analysis

The HS-GC-IMS system was used for subsequent analysis of LJF samples. LJF powder (1.0 g) was accurately weighed and placed into a 20 mL headspace vial. These samples were incubated at 80 °C for 15 min with an agitation rate of 500 rpm. After incubation, 100 μL of headspace gas was automatically injected into the 85 °C injector via a syringe. VOC separation was achieved by GC with a MXT-WAX capillary column (Restek Corporation, Bellefonte, PA, USA), and the column, GC, and IMS were all set to 60 °C. Nitrogen served as the carrier gas with a programmed flow. Detailed parameters see Table S6 for details. The total analysis time was 35 min. To determine VOC retention indices (RI), C_4_–C_9_ *n*-ketones were used as external references under the same chromatographic conditions as the samples. VOC identification relied on comparing drift time (Dt) and RI, utilizing VOCal 0.4.03 software, which accesses built-in NIST and IMS databases. RI parameters are provided by the NIST 2020 database, while Dt parameters are derived from the IMS database.

### 3.3. HS-SPME-GC-MS Analysis

HS-SPME was used for extracting VOCs, following this procedure: 1.0 g of finely powdered LJF sample was placed in a 20 mL headspace vial. The vial was pre-equilibrated at 80 °C for 15 min on a heated plate, after which a commercial fiber was inserted into the vial to extract VOCs for 30 min under identical conditions. Desorption of the fiber was performed in splitless mode at 250 °C for 5 min in the GC-MS injector. For VOC analysis, a GC-MS system with a DB-5 MS capillary column was employed. Chromatographic separation followed a column oven program.

Mass spectrometry was conducted in electron impact ionization mode. Carrier gas was helium at a flow rate of 1 mL/min. VOCs were characterized by comparison with the NIST 17-1 mass spectral database, with a similar threshold over 85%. Detailed parameters, including instrument information and fiber details, among others, are provided in Table S6.

### 3.4. UPLC-Q-TOF-MS Analysis

Each 150 mg aliquot of LJF powder was placed into a 5 mL Eppendorf tube and blended with 3 mL of a methanol/water (3:1, *v*/*v*) solution. The tube was then subjected to ultrasonic treatment in an ice-water bath for 30 min, followed by centrifugation of the supernatant at 12,000 rpm for 10 min. The supernatant was diluted with a 75% methanol/water solution at a 1:4 ratio, and 1.0 mL of the diluted supernatant was filtered through a 0.22 μm membrane filter prior to UPLC-Q-TOF-MS analysis.

Analysis was performed using a UHPLC system connected to a Q-TOF mass spectrometer with an ESI interface (Impact II, Bruker, Germany). Chromatographic separation was conducted on an Agilent ZORBAX SB C18 column (Agilent, Palo Alto, CA, USA) under the following conditions: column oven temperature 40 °C, injection chamber temperature 4 °C, and flow rate 0.3 mL/min. The mobile phase consisted of (A) 0.1% formic acid in water and (B) acetonitrile.

Mass spectrometry detection utilized an ESI source with a mass range of 50 to 1200 m/z. Detailed parameters, including instrument information, elution gradient, and MS parameters, are provided in Table S6.

### 3.5. Procedure for Quality Control (QC) Sample Preparation

For each analytical technique (HS-GC-IMS, HS-SPME-GC-MS, and UPLC-Q-TOF-MS), a QC sample was prepared by combining equal aliquots from each individual LJF sample. Each QC sample was then processed following the same extraction and analysis protocol detailed for individual samples in their respective sections (Section 3.2, Section 3.3 and Section 3.4).

### 3.6. DPPH Radical Scavenging Assay

The QC samples from each origin were used for the DPPH radical scavenging assay following the same extraction procedure (up to the centrifugation step in Section 3.4). The resulting supernatant was filtered through a 0.22 μm membrane and then concentrated by centrifugation to obtain the polysaccharides of LJF.

The polysaccharide content was determined according to the method of Zhao [49] with slight modifications. Briefly, solutions of polysaccharides at concentrations of 50, 100, 150, 200, 250, and 300 μg/mL were prepared for the assay.

### 3.7. Statistical Analysis

In this study, all experiments were repeated for triplicate. For assessing the significance of differences among LJF samples from different origins, SPSS 26.0 software was applied to carry out one-way analysis of variance (ANOVA). Results were shown as mean ± standard deviation. Pipe charts, heatmaps, Venn diagrams, and stacked bar charts were generated with Origin 2021 software. OPLS-DA was conducted using Simca 14.1 software. VOCal 0.4.03 software served for spectral analysis and characterization of HS-GC-IMS data. Two-dimensional topographic plots were created by using the Reporter plug-in. Moreover, the Gallery Plot plug-in was used to generate fingerprints.

## 4. Conclusions

In summary, this study characterized chemical profiles of LJF from four geographical origins, revealing that both VOCs and non-VOCs exhibit geographical specificity, which may be closely related to their environmental factors. VOCs such as *(E)*-2-octenal, *(E)*-2-heptenal, and benzaldehyde, as well as non-VOCs including cryptochlorogenic acid, luteolin, and sweroside, not only serve as key differential compounds but also contribute to diverse bioactivities (e.g., pest repellency, anti-microbial, and neuroprotective effects). The functional divergence was further supported by a DPPH antioxidant assay, where scavenging rates showed significant differences specifically at 100 and 300 μg/mL, with samples with an LY origin exhibiting the highest activity (Figure S5). Nevertheless, a limitation of this study is the lack of quantitative analysis. Future research should utilize reliable standards for the absolute quantification of key markers and integrate multiple bioactivity assays to further strengthen the connection between chemical disparities and functional results. Overall, the combined use of HS-GC-IMS, HS-SPME-GC-MS, and UPLC-Q-TOF-MS allowed for a more comprehensive characterization and deeper insight into the differences in chemical compounds among LJF from different origins.

## Data Availability

The original contributions presented in this study are included in the article/Supplementary Materials. Further inquiries can be directed to the corresponding author.

## Supplementary Materials

The following supporting information can be downloaded at: https://www.mdpi.com/article/10.3390/molecules31010004/s1, Figure S1: Dendrogram of the 62 *Lonicerae japonicae* flos based on SSR markers; Figure S2: The Comparative difference spectrum of two-dimensional topographic plots of QC samples by HS-GC-IMS; Figure S3: The chromatogram of QC samples by HS-SPME-GC-MSF; Figure S4: The total ion current chromatograms (TICs) of QC samples by UPLC-Q-TOF-MS. (A) Positive ion mode (ESI+). (B) Negative ion mode (ESI−); Figure S5: DPPH scavenging effect of polysaccharides from LJF; Table S1: Information about *Lonicerae japonicae* flos samples; Table S2: The information of identified volatile compounds by HS-GC-IMS; Table S3: The information of identified volatile compounds by HS-SPME-GC-MS; Table S4: The VIP value of key differential compounds by HS-GC-MS, HS-SPME-GC-MS, and UPLC-Q-TOF-MS; Table S5: The information of identified non-volatile compounds by UPLC-Q-TOF-MS; Table S6: The details of HS-GC-IMS, HS-SPME-GC-MS, and UPLC-Q-TOF-MS.
