# Supplementary material for: Differential Analysis of Non-Volatile and Volatile Organic Compounds in Lonicerae japonicae Flos Across Four Geographical Origins of China Using HS-GC-IMS, HS-SPME-GC-MS, UPLC-Q-TOF-MS, and Multivariate Statistical Methods
Source: Molecules. 2025 Dec 19;31(1):4. doi: 10.3390/molecules31010004 (PMC12787219; doi:10.3390/molecules31010004)
Supplement: Supplementary file 1 [file molecules-31-00004-s001.zip › molecules-4010279-supplementary.pdf]

Supporting Information

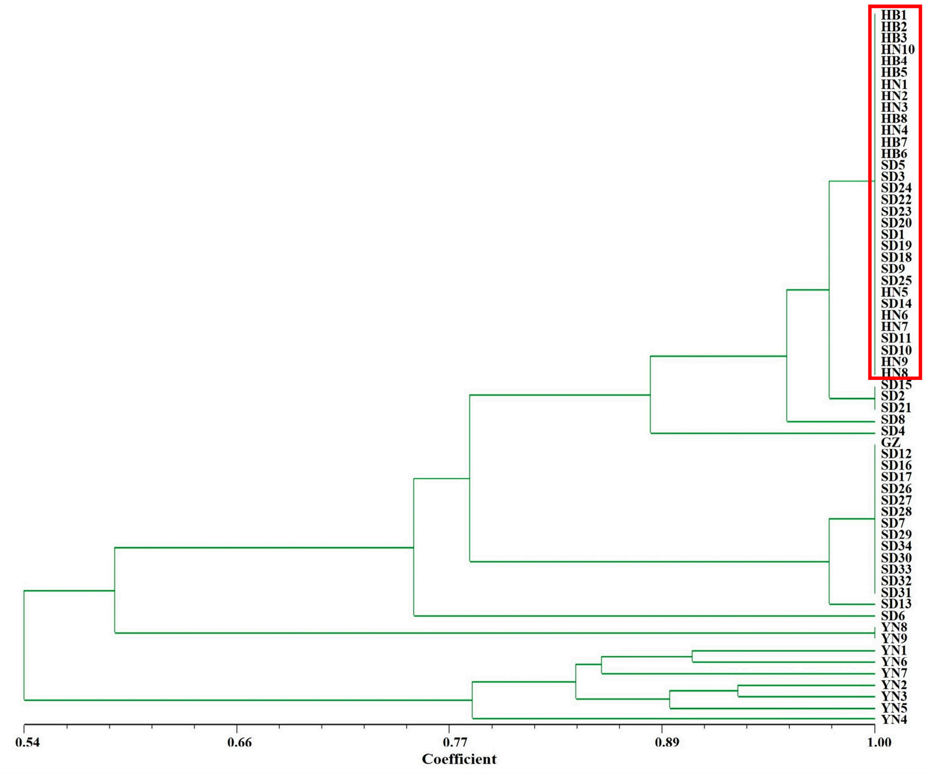

Figure S1. Dendrogram of the 62 *Lonicerae japonicae* flos based on SSR markers.

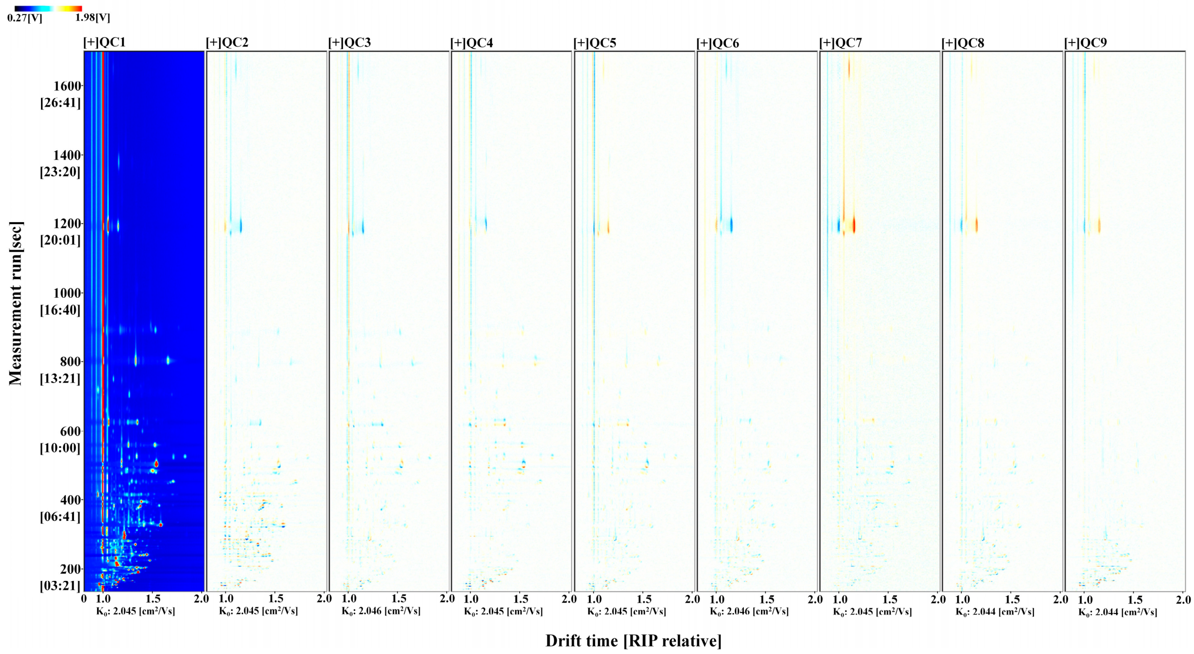

Figure S2. The Comparative difference spectrum of two-dimensional topographic plots of QC samples by HS-GC-IMS.

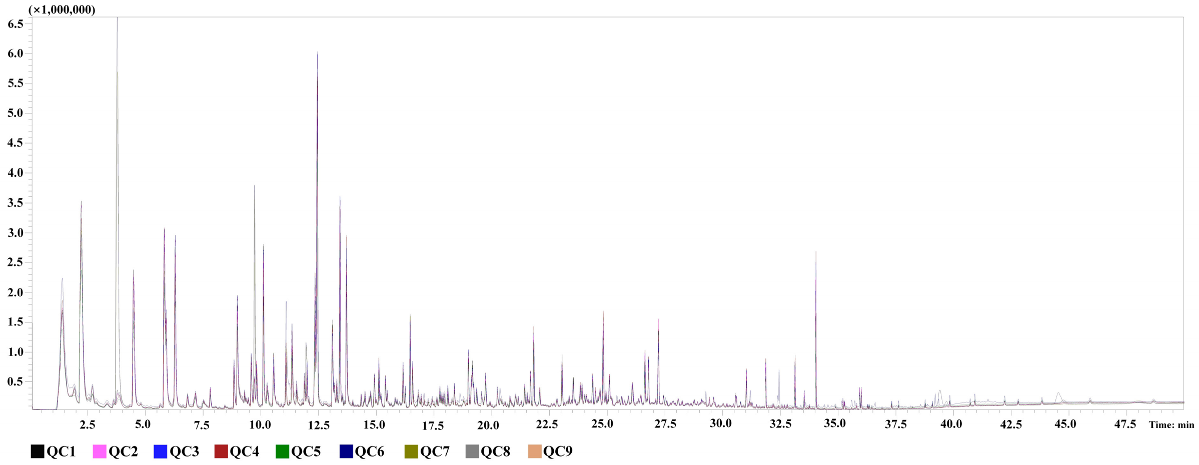

Figure S3. The chromatogram of QC samples by HS-SPME-GC-MS.

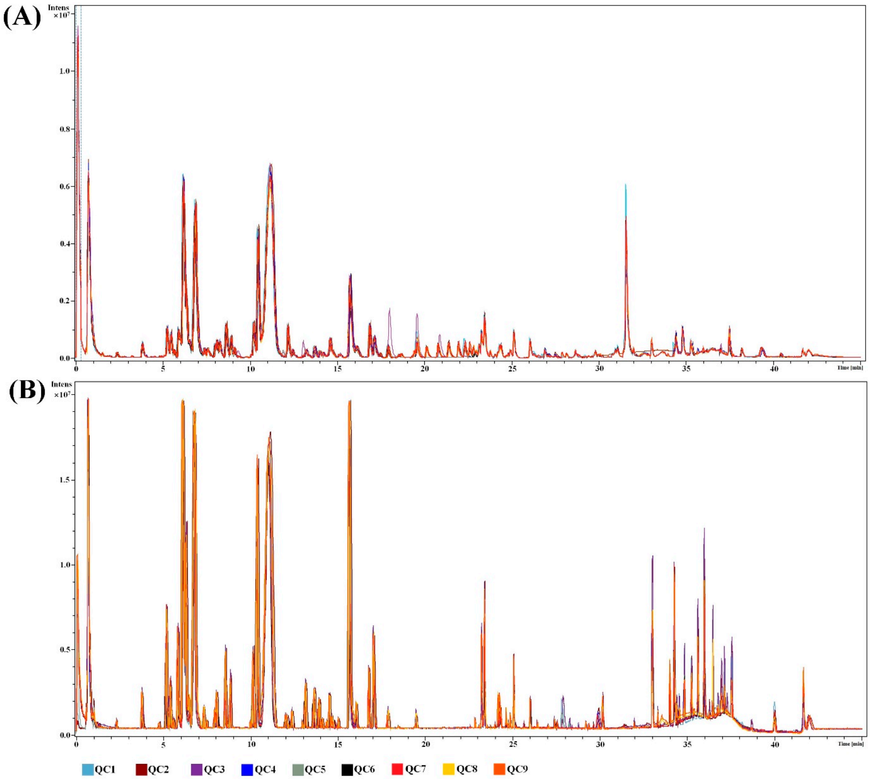

Figure S4. The total ion current chromatograms (TICs) of QC samples by UPLC-Q-TOF-MS. (A) Positive ion mode (ESI+). (B) Negative ion mode (ESI-).

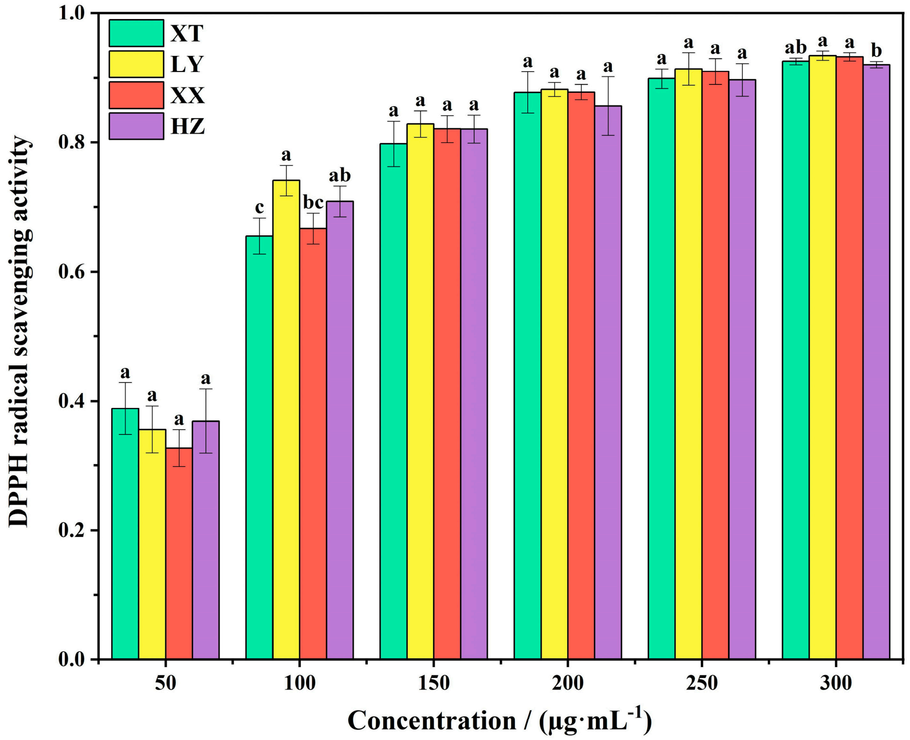

Figure S5. DPPH scavenging effect of polysaccharides from LJF.

Table S1. Information about *Lonicerae japonicae* flos samples.

Group	Previous number	Origin	Coordinate	Average annual temperature(℃) /sunshine time（h） /precipitation(mm)	Climate type
XX (XX1-XX10)	HN1-HN10	Xinxiang City, Henan Province, China	35.05°N, 114.42°E	14.2/2400/610	Temperate monsoon climate
XT (XT1-XX8)	HB1-HB8	Xingtai City, Hebei Province, China	37.23°N, 115.04°E	15.1/2407/532	Temperate monsoon climate
HZ (HZ1-HZ5)	SD3、SD5、SD9、SD10、SD11	Heze City, Shandong Province, China	35.58°N, 115.95°E	13.8/2388/585	Temperate monsoon climate
LY (LY1-LY9)	SD14、SD18-SD20、SD22-SD25、SD34	Linyi City, Shandong Province, China	35.52°N, 117.62°E	14.2/2200/826	Temperate monsoon climate

Table S2. The information of identified volatile compounds by HS-GC-IMS.

Classification	Compound	CAS	Formula	MW	RI	Rt [sec]	Dt [a.u.]	Peak volume			
								Group XX	Group XT	Group HZ	Group LY
**Aldehydes**	Benzaldehyde-M	100-52-7	C_7H_6O	106.1	1531.0	1376.314	1.15614	3121.0±2230.1a	1516.2±415.4b	2957.4±1250.8a	1838.8±383.9b
	Benzaldehyde-D	100-52-7	C_7H_6O	106.1	1528.9	1366.551	1.49038	2014.1±3528.0a	569.0±89.7a	1089.1±619.2a	608.0±57.1a
	(E)-2-Octenal	2548-87-0	$C_8H_{14}O$	126.2	1437.1	1000.118	1.34373	237.9±35.0○	315.1±111.6△	394.9±84.7b	607.6±93.6a
	(E)-2-Heptenal-M	18829-55-5	$C_7H_{12}O$	112.2	1330.7	696.429	1.26447	1141.0±342.6b	818.0±180.0△	1284.6±364.2b	2305.3±256.5a
	(E)-2-Heptenal-D	18829-55-5	$C_7H_{12}O$	112.2	1331.4	697.978	1.69357	405.3±219.5bc	213.3±58.2c	525.4±396.7b	1760.5±343.7a
	(E)-2-Hexen-1-al-M	6728-26-3	$C_6H_{10}O$	98.1	1230.7	499.961	1.18745	2267.8±106.4a	2250.3±267.3a	2079.9±130.0b	2200.4±163.4ab
	(E)-2-Hexen-1-al-D	6728-26-3	$C_6H_{10}O$	98.1	1229.3	497.757	1.53569	7877.1±418.9bc	8497.7±1787.2b	7470.0±378.5c	10259.2±1016.1a
	Heptaldehyde	111-71-7	$C_7H_{14}O$	114.2	1197.7	448.953	1.70226	1455.1±482.1b	1256.0±1093.0b	2771.6±742.2a	1157.1±157.9b
	(E)-2-Pentenal-M	1576-87-0	C_5H_8O	84.1	1146.8	378.057	1.10977	643.0±96.7a	610.8±78.3a	601.8±90.2a	491.5±82.2b
	(E)-2-Pentenal-D	1576-87-0	C_5H_8O	84.1	1146.2	377.294	1.37338	1071.7±206.1b	1525.5±225.3a	1099.6±46.5b	1429.2±117.9a
	1-Hexanal	66-25-1	$C_6H_{12}O$	100.2	1100.0	322.415	1.58081	3035.8±350.5c	3235.4±571.8bc	3927.8±490.4a	3529.6±352.8b
	n-Pentanal-D	110-62-3	$C_5H_{10}O$	86.1	1000.2	236.484	1.43918	278700.0±539.0a	2427.0±411.0ab	2527.4±516.3ab	2306.9±541.0b
	2-Methyl butanal	96-17-3	$C_5H_{10}O$	86.1	925.1	198.289	1.41649	1369.2±247.0a	1398.3±241.2a	1060.6±101.1b	1143.9±185.5b
	Butanal-M	123-72-8	C_4H_8O	72.1	885.7	181.065	1.11923	129.0±16.7a	102.3±14.4b	117.9±24.3a	96.3±23.5b
	Butanal-D	123-72-8	C_4H_8O	72.1	887.9	181.979	1.29176	754.7±163.3b	774.0±112.0ab	755.6±62.3b	851.6±67.5a
	Propanal	123-38-6	C_3H_6O	58.1	825.7	157.689	1.15105	1800.9±95.0b	1748.7±215.8b	2086.0±163.2a	2022.1±96.6a
	(Z)-2-Methylpent-2-enal-M	623-36-9	$C_6H_{10}O$	98.1	1163.2	399.671	1.16409	323.2±116.9c	408.1±77.6b	492.4±136.7a	391.1±64.9bc
	n-Pentanal-M	110-62-3	$C_5H_{10}O$	86.1	1005.5	240.425	1.18912	293.8±48.2a	278.2±44.8a	230.9±57.7b	237.9±26.6b
	3-Methyl-2-butenal	107-86-8	C_5H_8O	84.1	1211.5	469.692	1.09772	146.4±52.8a	139.6±40.5a	152.5±23.7a	145.9±290.0a
	1-Octanal	124-13-0	$C_8H_{16}O$	128.2	1300.0	627.319	1.41349	121.2±71.0b	109.6±17.8b	188.8±23.2a	164.0±32.6a
	2-Methyl-2-propenal	78-85-3	C_4H_6O	70.1	895.4	185.149	1.23470	75.4±18.8b	93.4±28.0b	87.5±27.8b	122.5±34.5a
	(E, E)-2,4-Hexadienal	142-83-6	C_6H_8O	96.1	1411.9	917.799	1.12103	240.9±38.4b	268.4±56.7b	294.7±43.0b	676.0±148.7a

	(Z)-2-Methylpent-2-enal-D	623-36-9	$C_6H_{10}O$	98.1	1163.6	400.231	1.50906	14900.0±73.5c	271.6±121.9b	416.4±217.7a	400.2±196.9a
**Ketones**	1-Hydroxy-2-propanone	116-09-6	$C_3H_6O_2$	74.1	1311.7	652.685	1.05317	446.5±76.2b	580.1±158.1a	569.7±68.1a	588.9±77.0a
	Acetoin-M	513-86-0	$C_4H_8O_2$	88.1	1296.5	619.968	1.06146	2757.8±223.3c	3320.3±263.8a	2647.6±96.8c	3143.3±76.3b
	Acetoin-D	513-86-0	$C_4H_8O_2$	88.1	1295.9	618.639	1.34403	1490.4±365.8b	3526.3±606.1a	1728.0±324.4b	3602.4±452.3a
	Isovalerone-M	108-83-8	$C_9H_{18}O$	142.2	1244.6	523.141	1.34901	226.4±127.8b	371.1±378.4b	845.6±535.5a	405.0±150.2b
	Isovalerone-D	108-83-8	$C_9H_{18}O$	142.2	1243.4	521.189	1.82663	49.7±18.0b	182.0±370.6b	453.8±348.3a	99.3±65.4b
	1-Penten-*3-O*ne-M	1629-58-9	C_5H_8O	84.1	1039.1	266.800	1.08073	324.4±122.6c	444.3±68.3b	496.1±77.3ab	537.1±67.4a
	1-Penten-*3-O*ne-D	1629-58-9	C_5H_8O	84.1	1038.2	266.010	1.32388	1063.0±138.8d	1565.1±122.5b	1403.4±119.1c	1957.4±202.8a
	2-Heptanone-M	110-43-0	$C_7H_{14}O$	114.2	1191.5	439.940	1.26858	431.6±152.2ab	420.5±61.3b	478.6±23.6ab	503.5±101.2a
	2-Heptanone-D	110-43-0	$C_7H_{14}O$	114.2	1192.7	441.665	1.65049	151.8±121.0bc	112.1±45.8c	206.4±41.3ab	247.8±115.2a
	2-Hexanone	591-78-6	$C_6H_{12}O$	100.2	1098.5	320.840	1.54503	245.4±79.9b	200.3±52.1b	383.0±69.0a	385.2±94.4a
	2-Pentanone	107-87-9	$C_5H_{10}O$	86.1	997.9	234.793	1.40783	707.3±109.1a	927.8±50.3a	869.3±118.9b	948.4±132.9b
	2-Butanone	78-93-3	C_4H_8O	72.1	911.5	192.140	1.25613	575.5±245.9b	1210.6±304.5a	641.8±120.9b	1332.8±316.7a
	2-Propanone	67-64-1	C_3H_6O	58.1	840.0	162.979	1.12617	1142.9±70.0c	1491.6±235.8b	1247.7±157.8c	1819.1±210.8a
	2-Methyl-2-hepten-6-one	110-93-0	$C_8H_{14}O$	126.2	1349.0	741.064	1.18771	457.5±244.0a	412.1±115.5a	463.1±84.0a	454.9±81.8a
	2,3-Pentadione	600-14-6	$C_5H_8O_2$	100.1	1076.3	299.325	1.22802	161.5±30.3b	245.2±63.2a	243.9±40.8a	262.4±23.2a
	3-Penten-2-one-M	625-33-2	C_5H_8O	84.1	1142.0	371.876	1.07877	288.4±69.4b	308.2±109.6b	333.3±29.5b	479.1±67.1a
	3-Penten-2-one-D	625-33-2	C_5H_8O	84.1	1142.1	372.027	1.35296	616.4±278.9c	873.6±3670.0bc	918.4±142.5b	2187.8±573.9a
**Alcohols**	1-Octen-3-ol	3391-86-4	$C_8H_{16}O$	128.2	1477.6	1147.702	1.17169	830.8±343.2c	550.3±186.1d	1100.8±255.0b	1848.7±295.3a
	(E)-2-Hexen-1-ol	928-95-0	$C_6H_{12}O$	100.2	1401.1	884.712	1.53135	3343.8±2587.6a	3969.8±989.9a	3329.4±1770.2a	2861.5±580.2a
	1-Hexanol-M	111-27-3	$C_6H_{14}O$	102.2	1369.6	794.715	1.33684	4231.4±423.6ab	4473.8±361.9a	4182.7±458.2b	4268.2±229.1ab
	1-Hexanol-D	111-27-3	$C_6H_{14}O$	102.2	1370.0	795.900	1.66252	2775.5±780.3b	3519.4±925.1a	3181.5±1067.0ab	3244.1±593.6ab
	1-Pentanol-M	71-41-0	$C_5H_{12}O$	88.1	1263.0	555.557	1.26014	2011.2±332.8b	2209.6±148.5a	2126.8±54.9ab	2148.8±151.2ab
	1-Pentanol-D	71-41-0	$C_5H_{12}O$	88.1	1261.8	553.480	1.52984	1634.7±700.8c	2324.6±694.1ab	2102.7±358.7bc	2701.4±817.2a
	2-Methyl-1-butanol-D	137-32-6	$C_5H_{12}O$	88.1	1217.1	478.261	1.50012	2916.2±838.1b	3892.0±413.5a	2790.5±487.3b	3175.5±631.8b
	1-Butanol-M	71-36-3	$C_4H_{10}O$	74.1	1156.0	389.972	1.18626	828.7±156.8a	811.3±71.7a	830.8±82.1a	675.8±101.7b

	1-Butanol-D	71-36-3	$C_4H_{10}O$	74.1	1156.1	390.132	1.39220	466.6±177.0b	604.9±136.1a	583.9±62.1a	668.6±134.7a
	1-Propanol-M	71-23-8	C_3H_8O	60.1	1049.6	275.558	1.11319	510.7±199.3c	613.9±133.4bc	694.5±160.3ab	732.5±94.6a
	1-Propanol-D	71-23-8	C_3H_8O	60.1	1050.1	276.058	1.25862	357.7±270.6c	673.6±322.8b	925.2±362.4a	1026.6±328.7a
	Ethanol	64-17-5	C_2H_6O	46.1	946.8	208.427	1.14044	2583.4±410.5c	3546.4±610.9b	3642.9±969.9b	4183.7±412.8a
	2-Methyl-2-propanol	75-65-0	$C_4H_{10}O$	74.1	919.2	195.604	1.33463	524.5±112.9b	378.7±194.3c	863.4±123.5a	319.7±63.6c
	2-Methyl-1-butanol-M	137-32-6	$C_5H_{12}O$	88.1	1217.3	478.602	1.23906	763.8±101.4a	614.2±89.3b	725.3±27.8a	550.8±69.5c
	3-Methyl-1-butanol	123-51-3	$C_5H_{12}O$	88.1	1218.3	480.224	1.25369	736.4±134.2a	727.0±53.5a	699.1±25.2ab	636.3±91.3b
	3-Pentanol	584-02-1	$C_5H_{12}O$	88.1	1106.3	329.440	1.42906	329.4±70.4c	575.5±181.7a	360.0±47.3bc	441.2±118.2b
**Esters**	Ethyl propanoate	105-37-3	$C_5H_{10}O_2$	102.1	970.1	219.917	1.46947	59.3±43.3b	161.0±318.4b	552.4±408.4a	117.7±58.8b
	Acetic acid ethyl ester	141-78-6	$C_4H_8O_2$	88.1	895.6	185.269	1.35297	2405.8±293.1a	2234.6±418.0ab	2331.8±572.6a	2020.8±434.0b
	Methyl acetate	79-20-9	$C_3H_6O_2$	74.1	854.1	168.347	1.20434	2948.4±367.9a	2512.7±535.5b	3265.3±542.6a	1811.2±371.7c
	Amyl acetate	628-63-7	$C_7H_{14}O_2$	130.2	1184.0	428.849	1.32320	269.3±118.3ab	242.7±120.3bc	333.0±55.4a	193.1±58.8c
	Acetic acid hexyl ester-M	142-92-7	$C_8H_{16}O_2$	144.2	1283.4	593.772	1.39581	527.2±164.1ab	565.5±200.8a	652.4±358.9a	378.5±79.6b
	Ethyl isobutyrate	97-62-1	$C_6H_{12}O_2$	116.2	990.7	230.585	1.21418	62.3±25.8c	107.3±29.4a	86.1±34.9b	122.1±17.2a
	Acetic acid hexyl ester-D	142-92-7	$C_8H_{16}O_2$	144.2	1284.5	595.850	1.92482	78.2±25.6b	87.5±39.9b	148.0±131.9a	51.8±11.6b
	Methyl isovalerate-D	556-24-1	$C_6H_{12}O_2$	116.2	1023.7	254.338	1.55280	181.6±218.4b	109.6±173.0b	554.1±306.8a	98.2±63.2b
	Ethyl acrylate	140-88-5	$C_5H_8O_2$	100.1	1023.9	254.513	1.42922	49.1±13.2b	107.6±51.6a	68.4±14.0b	128.5±42.9a
	Acetic acid propyl ester	109-60-4	$C_5H_{10}O_2$	102.1	990.7	230.603	1.17352	380.6±37.6a	341.5±50.2ab	333.2±117.9ab	323.1±70.9b
	Isoamyl acetate-M	123-92-2	$C_7H_{14}O_2$	130.2	1134.0	361.908	1.31361	323.8±187.9a	336.0±127.5a	305.2±152.9a	190.2±73.9b
	Isoamyl acetate-D	123-92-2	$C_7H_{14}O_2$	130.2	1133.4	361.147	1.76941	169.9±230.7a	199.6±167.9a	154.0±129.3a	100.9±49.3a
	Methyl isovalerate-M	556-24-1	$C_6H_{12}O_2$	116.2	1021.7	252.768	1.20015	150.5±34.3b	140.3±32.8b	214.9±65.9a	134.5±34.8b
**Terpenoids**	Linalool	78-70-6	$C_{10}H_{18}O$	154.3	1589.9	1681.398	1.23303	667.6±458.7a	457.1±99.6b	737.0±139.7a	430.5±60.6b
	Limonene	138-86-3	$C_{10}H_{16}$	136.2	1198.4	449.931	1.29452	977.1±123.9a	738.4±187.8b	1033.0±69.6a	651.5±69.1b
	β-Ocimene	13877-91-3	$C_{10}H_{16}$	136.2	1240.2	515.698	1.26376	340.9±117.7b	416.0±266.3b	707.4±364.3a	632.3±120.1a
	β-Pinene-M	127-91-3	$C_{10}H_{16}$	136.2	1118.7	343.547	1.22611	174.6±38.2a	127.6±40.4b	107.2±20.0b	136.5±63.7b
	β-Pinene-D	127-91-3	$C_{10}H_{16}$	136.2	1119.9	344.945	1.30593	109.6±21.1a	109.8±14.8a	107.4±13.0a	86.1±8.4b

**Acids**	2-Methylpropanoic acid	79-31-2	$C_4H_8O_2$	88.1	1627.3	1909.306	1.16560	1565.9±296.3c	1101.2±236.9d	2122.6±543.1b	3056.8±824.6a
	Acetic acid-M	64-19-7	$C_2H_4O_2$	60.1	1487.7	1187.890	1.05560	15647.0±1960.8a	11103.6±2573.4b	15765.0±1135.9a	11551.0±1620.9b
	Acetic acid-D	64-19-7	$C_2H_4O_2$	60.1	1486.5	1182.955	1.16227	1657.2±340.2b	892.9±449.3d	2447.9±335.7a	1271.6±427.2c
	Propanoic acid	79-09-4	$C_3H_6O_2$	74.1	1583.2	1643.665	1.11165	1117.3±335.3b	516.4±324.2c	1617.4±302.0a	565.8±140.0c
**Furans**	2-Ethyl furan	3208-16-0	C_6H_8O	96.1	969.2	219.474	1.04945	1574.1±123.8c	1716.9±206.1b	1562.2±161.8c	2107.7±198.8a
**Unidentified**	1	unidentified	a	0	1112.6	336.588	1.36410	791.5±704.5ab	993.1±4860.0ab	1102.3±304.7a	714.3±381.4b
	2	unidentified	a	0	1113.8	337.881	1.09670	391.6±79.7a	338.3±49.6b	385.5±74.6a	200.1±27.3c
	3	unidentified	a	0	1060.5	285.067	1.21577	5771.0±492.2a	5608.2±380.0ab	5462.2±242.5b	5654.1±227.7ab
	4	unidentified	a	0	997.1	234.241	1.37790	485.0±185.9c	1212.6±232.8ab	913.7±377.3b	1497.6±617.1a
	5	unidentified	a	0	915.8	194.060	1.29488	641.5±108.4b	504.3±237.0c	869.5±118.8a	442.6±41.6c
	6	unidentified	a	0	835.7	161.376	1.29124	667.7±60.9a	410.6±124.6c	560.2±69.8b	348.4±117.3c
	7	unidentified	a	0	1275.9	579.500	1.07781	161.5±124.6a	59.5±10.6b	163.0±40.5a	64.4±12.0b
	8	unidentified	a	0	1104.0	326.902	1.49300	573.2±121.9d	951.4±141.2d	696.5±77.8c	1060.6±142.7a
	9	unidentified	a	0	964.1	216.898	1.19791	208.4±35.2a	127.6±34.8c	172.5±37.9b	201.5±41.1a
	10	unidentified	a	0	802.8	149.616	1.13043	204.0±60.2c	790.7±145.1a	332.0±110.1b	703.8±130.4a
	11	unidentified	a	0	866.5	173.253	1.06494	244.2±56.9b	276.5±109.9b	161.8±56.4c	443.1±80.3a
	12	unidentified	a	0	1110.1	333.686	1.39881	76.9±16.3c	128.1±44.9a	98.7±17.2bc	117.2±27.5ab
	13	unidentified	a	0	1142.9	372.988	1.43881	71.4±21.7c	93.6±21.1ab	81.9±8.2bc	102.4±14.7a
	14	unidentified	a	0	1084.0	306.569	1.43249	274.3±118.7b	189.3±80.7c	353.4±101.5b	637.8±116.0a
	15	unidentified	a	0	1010.5	244.174	1.32799	330.9±164.3b	397.7±154.1b	288.8±1150.0b	897.7±420.3a
	16	unidentified	a	0	1063.0	287.321	1.47293	37.5±31.1c	63.5±65.8bc	101.5±48.7a	83.7±27.8ab

Note: MW: Molecular weight; RI: Retention index; Rt: Retention time; Dt: Drift time; Suffix M represented the monomer of volatile compound and suffix D represented the dimer of volatile compound; Values with different letters in a row indicated significant differences using analysis of variance (ANOVA) ($p < 0.05$).

Table S3. The information of identified volatile compounds by HS-SPME-GC-MS.

Classification	Compounds	RT	CAS	Peak areas			
				Group XX	Group XT	Group HZ	Group LY
**Aldehydes**	*(E)*-2-Decenal	18.392	3913-81-3	371560.5±110661.5ab	188721.4±159858.4c	286847.2±164396.1bc	408433.8±174520.0a
	(E)-2-Dodecenal	21.180	20407-84-5	n.d.	255719.5±171242.8a	n.d.	151309.4±47444.4b
	trans-2-Heptenal	9.129	18829-55-5	1655365.8±187677.4a	525899.2±117029.3c	1137074.5±445782.7b	1659187.9±651086.5a
	**2-Hexenal**	6.067	505-57-7	n.d.	2831893.2±1778346.3b	1449813.3±1087367.7bc	**6763536.9±4994779.6a**
	***trans*-2-Hexenal**	6.073	6728-26-3	149795.6±105742.9b	477193.4±220657.0b	215447.7±58234.5b	**3985145.8±6134405.7a**
	(E)-2-Octenal	12.292	2548-87-0	778739.4±126766.8b	803574.4±234618.6b	664922.3±213216.2b	1050795.3±175910.5a
	2-Butyl-2-octenal	21.342	13019-16-4	217926.4±614919.3a	248421.4±260807.9a	96518.6±123189.6a	n.d.
	(E)-2-Pentenal	3.820	1576-87-0	n.d.	n.d.	54109.3±69730.2b	166538.6±112078.3a
	**Benzaldehyde**	9.265	100-52-7	**20514817.7±4305891.8a**	**15440340.8±1838520.6b**	**9176421.1±7075897.8c**	**4890925.0±1425996.8d**
	Benzene acetaldehyde	11.831	122-78-1	528827.9±67663.8b	554293.4±43973.9b	563513.3±307526.7b	694333.9±197775.6a
	Decanal	16.762	112-31-2	1606895.7±235185.2b	2090954.5±389235.4a	1553967.0±598393.8b	2199047.9±432248.9a
	Dodecanal	22.377	112-54-9	506077.1±233926.3a	649872.2±151004.3a	310101.1±300049.6b	524994.1±139124.5a
	Heptanal	7.439	111-71-7	n.d.	480587.5±227930.8b	n.d.	752059.0±253715.5a
	**Hexanal**	4.709	66-25-1	**6348109.6±885346.4b**	**9741683.8±3968876.1a**	**4112030.1±570304.9c**	**11238755.0±2641457.1a**
	Lilac aldehyde B	15.498	53447-45-3	1412799.2±351782.4a	662085.3±156740.1c	863875.0±202850.8b	789983.1±162399.6bc
	**Nonanal**	13.722	124-19-6	3195405.9±396878.1c	**4123719.9±559252.4b**	**2659923.2±785288.3d**	**5456280.2±734445.6a**
	Octanal	10.560	124-13-0	183974.7±189830.8c	526901.4±165841.8b	224558.9±262217.2c	1197235.3±234028.5a
	Pentanal	2.879	110-62-3	1736492.9±730676.0a	1641916.4±377747.8a	1070306.2±224609.7b	n.d.
	Tetradecanal	22.397	124-25-4	57986.3±76688.7b	278062.4±265818.6a	77547.2±21362.1b	36059.0±42684.9b
	Tridecanal	19.889	10486-19-8	26689.6±37577.6c	138751.5±41017.5a	117187.3±61020.3ab	87686.3±31305.1b
**Alcohols**	2-Methyl-1-butanol	3.499	137-32-6	168311.5±162890.6b	552841.0±203865.0a	n.d.	n.d.
	Isoamyl alcohol	3.518	123-51-3	195824.7±305229.1b	601483.5±288933.0a	n.d.	n.d.
	**1-Hexanol**	6.520	111-27-3	**6571185.5±953033.0ab**	**7451750.0±514385.5a**	**6153894.6±1122197.6b**	**6432317.7±1898185.6b**
	2-Ethylhexanol	11.374	104-76-7	862974.3±515225.7a	1001507.2±638684.7a	1012554.7±968215.8a	1050650.8±264162.3a

	**1-Octanol**	12.715	111-87-5	**5875164.6±4270419.3b**	**11936779.4±797302.3a**	**10939186.7±2275567.0a**	**9587325.2±3442031.8a**
	**1-Octen-3-ol**	9.848	3391-86-4	**4041804.7±612456.0a**	2550017.7±2021843.7c	**2930399.7±625580.1bc**	**3509362.6±1005798.5ab**
	1-Pentanol	4.063	71-41-0	559598.0±312251.3b	863244.3±478243.7a	178621.6±73542.1c	833607.6±226571.4a
	trans-2-Hexen-1-ol	6.455	928-95-0	n.d.	n.d.	n.d.	90148.8±32778.6a
	**3-Hexen-1-ol**	6.106	544-12-7	**7226923.4±2921317.9b**	**11472851.7±3709201.7a**	n.d.	n.d.
	***cis*-3-Hexen-1-ol**	6.124	928-96-1	**4509289.2±5995890.2a**	0.0±0.0b	**2749441.2±3250363.8a**	n.d.
	cis-3-Octen-1-ol	12.165	20125-84-2	1339523.7±569109.5b	2863803.0±886476.2a	347620.8±445211.4c	309252.9±271571.0c
	**Benzyl alcohol**	11.581	100-51-6	**3326536.5±999924.8a**	**3436373.5±1128229.1a**	2348518.2±787641.2b	1525596.1±414908.9c
	1-Nonadecanol	35.328	1454-84-8	8435.6±25825.2b	277930.6±164468.9a	n.d.	28517.3±82257.3b
	**2-Phenylethanol**	13.997	1960/12/8	**7786126.7±1134171.0a**	**7528418.1±1981959.2a**	**7220275.0±2076339.6a**	**5535203.8±972898.7b**
**Alkanes**	3,8-Dimethyldecane	17.890	17312-55-9	96749.9±148392.6a	n.d.	36072.2±46071.6ab	59127.0±113071.3ab
	Dodecane	16.569	112-40-3	1453762.6±191217.8a	928075.7±374831.1b	573106.4±324587.4c	899208.6±122980.5b
	2,7,10-Trimethyldodecane	20.774	74645-98-0	48755.5±100369.5ab	n.d.	101423.5±217486.3a	41006.7±78620.9ab
	4,6-Dimethyldodecane	19.300	61141-72-8	250410.1±176218.6a	171052.7±83869.6a	193934.5±137041.4a	163775.9±215343.2a
	Heneicosane	37.499	629-94-7	274323.0±122403.4a	324562.3±106399.6a	173230.2±62001.8b	316637.7±139196.8a
	Hexadecane	27.100	544-76-3	581506.5±375472.4b	1088446.5±280730.6a	658080.3±501276.1b	619659.2±355376.8b
	Nonadecane	17.723	629-92-5	930875.3±1102722.8a	n.d.	993150.7±864076.8a	1081774.4±258314.0a
	3-Methyl-5-propylnonane	18.938	31081-18-2	35200.0±77585.2c	146989.1±44097.4a	89013.0±93710.3b	n.d.
	Octadecane	31.418	593-45-3	208659.8±205221.1ab	283301.7±127553.7a	120511.2±31144.8b	151791.8±78457.4b
	Pentacosane	39.248	629-99-2	126091.0±31104.5a	161471.1±40339.8a	126316.7±67308.6a	131399.8±67703.1a
	Tetradecane	22.137	629-59-4	2055276.6±1201188.0b	2773953.6±333835.4a	1185163.7±866107.4c	2776767.4±560218.5a
	Tridecane	16.565	629-50-5	n.d.	1893492.0±159465.5a	n.d.	n.d.
**Esters**	*(E)*-3-Hexenyl acetate	10.596	3681-82-1	1399295.3±347371.2a	n.d.	718598.2±460436.7b	n.d.
	cis-3-Hexenyl acetate	10.608	3681-71-8	298064.8±306979.0a	437027.7±320571.8a	271300.7±234969.6ab	81373.0±234818.5b
	cis-3-Hexenyl benzoate	26.426	25152-85-6	1798227.6±886614.8a	1433393.2±1029790.3a	713634.1±427446.3b	500930.9±726011.1b
	Hexyl acetate	10.833	142-92-7	1777611.3±329081.3ab	2836864.6±4129026.6a	1651504.4±739666.8ab	312955.8±488723.6b
	Hexyl benzoate	26.608	6789-88-4	267950.6±118598.4a	292360.0±259471.9a	127437.1±24705.5b	291929.4±130364.5a
	Benzyl benzoate	30.835	120-51-4	858223.6±190069.2a	751623.5±151541.1a	715127.2±400530.6a	518799.2±206184.7b

	Dibutyl phthalate	33.755	84-74-2	n.d.	280195.1±405784.8a	127802.0±96093.5ab	62218.4±69391.1b
	Ethyl palmitate	34.237	628-97-7	196804.0±95925.4b	163776.6±63097.7b	167075.8±82656.8b	333795.6±154572.2a
	**Methyl palmitate**	33.350	112-39-0	3102019.4±963235.0a	2367399.7±868613.6b	**2440204.0±1327706.0ab**	1165852.6±508464.1c
	(Z)-3-Hexenyl hexanoate	21.585	31501-11-8	452375.7±112224.6b	708873.1±485891.4a	190890.1±59532.4c	199795.9±50108.4c
	Methyl stearate	35.768	112-61-8	59158.1±39877.2a	85518.5±61572.0a	73468.3±10934.5a	n.d.
	Methyl tetradecanoate	29.912	124-10-7	892055.5±369872.0a	853255.3±267680.8a	536761.2±319288.4b	350878.5±144741.6b
**Ketones**	1-Octen-3-One	9.736	4312-99-6	125555.1±14898.1b	93639.6±59543.5b	143011.4±37038.4b	501953.9±140702.3a
	2-Heptadecanone	33.013	2922-51-2	286368.4±117747.3a	261312.0±69826.6a	211428.0±144722.5a	63491.5±67783.3b
	2-Heptanone	7.071	110-43-0	n.d.	735061.2±481715.9a	367095.1±182745.8b	198905.2±237993.0bc
	2-Nonanone	13.293	821-55-6	22436.0±45854.4b	222661.6±206963.2a	60497.4±52414.2b	38442.4±56561.8b
	**3,5-Octadien-2-one**	13.397	38284-27-4	**6518173.0±1470225.6a**	**6110643.5±2942553.7a**	**3540019.3±587417.2b**	**3343622.2±1428544.9b**
	5,6-Epoxy-*β*-ionone	24.238	23267-57-4	1341600.8±953096.8a	n.d.	978298.0±286645.1ab	746415.7±117415.9b
	2-Methyl-*3-O*ctanone	9.985	923-28-4	702393.0±745129.6a	486668.0±398950.7ab	440720.4±379114.0ab	150004.0±432466.4b
	**3-Octen-2-one**	11.642	1669-44-9	1254015.6±481534.3b	**3091804.7±3237790.8a**	761306.8±425773.7b	1927109.0±453003.7ab
	6-Methyl-5-hepten-2-one	9.965	110-93-0	843468.1±720497.4a	1273106.3±836818.0a	352099.6±320999.8b	n.d.
	2,2,6-Trimethylcyclohexanone	11.505	2408-37-9	37769.1±50708.4a	n.d.	42672.5±54720.4a	45417.0±57202.7a
**Terpenoids**	*α*-Terpineol	16.449	98-55-5	319526.6±177811.3a	n.d.	171021.5±40738.4b	n.d.
	trans-Nerolidol	26.171	40716-66-3	n.d.	287490.8±103634.1a	n.d.	n.d.
	β-Cyclocitral	17.821	432-25-7	131639.7±100395.9b	828449.0±141204.4a	n.d.	n.d.
	Spathulenol	26.588	6750-60-3	n.d.	242102.4±114423.0a	n.d.	n.d.
	Jasmone	21.947	488-10-8	n.d.	786033.2±434162.7a	n.d.	n.d.
	6,10-Dimethyl-5,9-undecadien-2-one	23.341	689-67-8	2155235.5±422817.0a	2339710.9±467938.6a	1207915.8±463214.5b	1152921.9±205596.3b
	Geraniol	18.055	106-24-1	899084.1±126213.1a	1167204.7±252744.5a	600259.9±78365.1a	910648.3±1519553.1a
	Linalool	13.576	78-70-6	2028827.0±911741.3a	2047659.0±745706.8a	1082431.8±529146.0b	1707234.3±338847.4a
	Phytol	26.329	150-86-7	31380.3±42225.9ab	n.d.	20608.8±42671.4bc	47238.2±31407.7a
	β-Ionone	24.139	79-77-6	1071275.8±936078.8a	n.d.	134375.2±279087.1b	330350.9±486062.4b
	trans-Linalool oxide (furanoid)	13.141	34995-77-2	242141.3±47368.8a	239328.0±73628.7a	96402.5±18497.3c	137458.4±30312.8b
**Furans**	2-Pentylfuran	10.114	3777-69-3	1416196.1±1904934.7b	1102205.7±655338.1b	1034009.3±253699.5b	2387365.9±394823.9a

Note: RI: Retention index; n.d.: Not detected; Values with different letters in a row indicated significant differences using analysis of variance (ANOVA) ($p < 0.05$); Boldface entries denote the top 10 compounds for each origin.

Table S4. The VIP value of key differential compounds by HS-GC-MS, HS-SPME-GC-MS, and UPLC-Q-TOF-MS. (Variable Importance in Projection (VIP) values from the OPLS-DA model.)

	Compound	VIP value
HS-GC-IMS	*(E)*-2-Heptenal	1.43
	2-Methyl-2-propanol	1.41
	1-Octen-3-ol	1.39
	Acetoin	1.31
	(E, E)-2,4-Hexadienal	1.30
	Propanoic acid	1.30
	2-Methylpropanoic acid	1.27
	Heptaldehyde	1.24
	1-Penten-3-one	1.24
	2-Hexanone	1.23
	Methyl acetate	1.22
	(E)-2-Octenal	1.20
HS-SPME-GC-MS	Dodecane	1.46
	Nonanal	1.41
	Tridecane	1.41
	(E)-3-Hexenyl acetate	1.39
	β-Cyclocitral	1.37
	trans-2-Hexen-1-ol	1.35
	Octanal	1.34
	Heptanal	1.33
	3-Hexen-1-ol	1.33
	trans-Nerolidol	1.32
	Benzaldehyde	1.32
	Tridecanal	1.31
	trans-Linalool oxide (furanoid)	1.30
	1-Octen-3-one	1.29
	Spathulenol	1.25

	Lilac aldehyde B	1.24
	2-Methyl-1-butanol	1.24
	cis-3-Octen-1-ol	1.23
	α-Terpineol	1.22
	Jasmone	1.22
	6,10-Dimethyl-5,9-undecadien-2-one	1.22
	Hexanal	1.22
UPLC-Q-TOF-MS	Diosmetin	1.40
	FA 16:1	1.38
	Arbutoside I	1.37
	Diosmetin-Oglucoside II	1.36
	Cryptochlorogenic acid	1.35
	Luteolin	1.33
	Arbutoside II	1.33
	4-O-Feruloylquinic acid	1.29
	Lonijaposide T	1.27
	Isoeugenol	1.27
	Tryptophan	1.25
	Dimethylsecologanoside I	1.24
	Serine	1.22
	Diosmetin-Oglucoside I	1.22
	Secologanoside III	1.21
	3-5-O-caffeoylquinic acid-glucoside	1.21
	Quercetin-*3-O*-glucoside	1.21
	Caffeic acid	1.20
	Sweroside	1.20

Table S5. The information of identified non-volatile compounds by UPLC-Q-TOF-MS.

Classification	Compounds	Formula	Mode	RT	m/z	Peak areas			
						Group XX	Group XT	Group HZ	Group LY
**Flavonoids**	Apigenin-*5*-*O*-neohesperidoside	$C_{27}H_{30}O_{14}$	M-H	15.7	577.1563	70450.1±13655.8c	100125.6±19984.6a	78775.2±9147.2bc	84753.9±10872.2b
	Apigenin-*7*-*O*-neohesperidoside	$C_{27}H_{30}O_{14}$	M-H	16.5	577.1563	388523.4±101494.5b	530754.4±170725.7a	417632.7±34910.4b	352949.4±44186.2b
	Apigenin-7-glucoside	$C_{21}H_{20}O_{10}$	M-H	16.4	431.0984	571088.6±190389.8c	1241554.1±558444.4a	951712.7±94633.7b	771723.8±181031.1bc
	Isorhamnetin-*3*-*O*-glucoside	$C_{22}H_{22}O_{12}$	M-H	16.4	477.1039	806434.3±200000.8c	1102376.6±135743.9bc	1532405.4±605237.4ab	2030107.2±1215869.7a
	Luteoloside	$C_{15}H_{10}O_{6}$	M-H	14.1	447.0933	11075054.2±3048236.2c	18261833.3±5360892.5a	13993937.4±983231.3b	13498279.4±1787834.1b
	Quercetin-*3*-*O*-glucoside	$C_{21}H_{20}O_{12}$	M-H	13.8	463.0882	10578839.2±2887355.1b	9638651.1±1268492.1b	9835278.7±751452.4b	13565766.1±1843221.1a
	Isorhamnetin-*O*-rutinoside I	$C_{28}H_{32}O_{16}$	M-H	15.8	623.1618	855185.8±195895.6b	1300316.6±345962.4a	803165.2±236906.4b	1163965.2±216570.8a
	Isorhamnetin-*O*-rutinoside II	$C_{28}H_{32}O_{16}$	M-H	16.4	623.1618	42603.2±12854.7bc	62494.6±22903.7a	51351.4±7540.1b	37292.1±4212.5c
	Kaempferol-*O*-rutinoside I	$C_{27}H_{30}O_{15}$	M-H	13.7	593.1512	1538854.9±357059.1b	1962610.6±406376.0a	1479080.1±220625.9b	1824048.0±211897.1a
	Kaempferol-*O*-rutinoside II	$C_{27}H_{30}O_{15}$	M-H	14.5	593.1512	4738358.6±1115802.3b	5313550.7±1100814.3a	4363035.7±413308.0b	4415344.9±393527.8b
	kaempferol-*O*-rutinoside III	$C_{27}H_{30}O_{15}$	M-H	15.3	593.1512	3321906.6±821759.8a	3353962.1±675982.1a	2508323.1±831056.3b	3240023.6±746671.5a
	Quercetin-*O*-diglucoside	$C_{27}H_{30}O_{17}$	M-H	7.6	625.1410	1561254.5±363694.5b	1906143.6±394118.8a	1567106.1±77422.9b	2006790.9±208530.0a
	Rutin	$C_{27}H_{30}O_{16}$	M-H	13.3	609.1461	20174664.5±4783313.0b	23705580.9±5035545.1a	17386453.3±4276774.7b	23875073.4±3768057.6a
	Diosmin	$C_{28}H_{32}O_{15}$	M-H	17.2	607.1668	218913.7±50958.6b	262873.2±57474.2b	328061.0±118416.7a	239661.8±53126.9b
	Luteolin	$C_{15}H_{10}O_{6}$	M-H	21.5	285.0405	1057271.4±374069.5c	931518.0±414173.0c	1933665.4±264440.5a	1411585.4±214459.6b
	Kaempferol-*O*-acetylglucoside	$C_{23}H_{22}O_{12}$	M-H	17.4	489.1039	45585.3±18847.0b	60534.8±8991.1b	73285.1±26560.2b	121878.2±77196.1a
	Quercetin-*O*-acetylglucoside	$C_{23}H_{22}O_{13}$	M-H	15.0	505.0988	85441.5±32359.1b	146791.2±22031.7b	223410.6±135623.9a	279981.3±136598.4a
	Quercetin-*O*-malonylglucoside	$C_{24}H_{22}O_{15}$	M-H	15.0	549.0886	72239.4±26341.2b	116023.0±16295.0b	175243.6±97804.9a	222722.0±105708.7a
	Diosmetin	$C_{16}H_{12}O_{6}$	M-H	24.2	299.0561	148521.5±34919.2b	150240.6±45822.8b	276559.0±67952.6a	172701.2±42556.3b
**Phenolic acids**	Neochlorogenic acid	$C_{16}H_{18}O_{9}$	M-H	3.9	353.0878	10762358.2±2662068.5ab	9367168.0±1902172.9c	9887377.8±1872756.0bc	11744932.3±1383324.6a
	Chlorogenic acid	$C_{16}H_{18}O_{9}$	M-H	6.3	353.0878	177229057.9±36391804.9c	189404338.0±9482404.1ab	179780792.5±11374972.4bc	193185921.5±14137938.7a
	Cryptochlorogenic acid	$C_{16}H_{18}O_{9}$	M-H	8.2	353.0878	4799208.6±2061237.8c	9683122.9±1181683.3b	7297646.0±4263854.0bc	25138516.6±7050757.5a
	3-*O*-*p*-Coumaroylquinic acid	$C_{16}H_{18}O_{8}$	M-H	8.6	337.0929	2301679.9±525475.4b	2361664.9±390651.7b	2756559.0±197287.1a	2890240.5±339629.0a
	4-*O*-*p*-Coumaroylquinic acid	$C_{16}H_{18}O_{8}$	M-H	10.5	337.0929	410560.6±98760.0b	491819.4±57608.2a	377651.6±91367.0bc	339989.1±53767.0c

	3-O-Feruloylquinic acid	$C_{17}H_{20}O_9$	M-H	10.1	367.1035	5484751.2±1384068.5a	5400443.7±703851.8a	5242518.1±1263595.2a	4987003.2±533447.9a
	4-*O*-Feruloylquinic acid	$C_{17}H_{20}O_9$	M-H	11.8	367.1035	423510.9±116983.0b	161965.0±29011.5c	749451.1±316159.8a	290806.7±207795.0bc
	Isochlorogenic acid B	$C_{25}H_{24}O_{12}$	M-H	15.2	515.1195	6045851.7±1476682.2a	5096028.1±1445872.4bc	5323299.7±1633865.3ab	4272485.0±730538.1c
	Isochlorogenic acid A	$C_{25}H_{24}O_{13}$	M-H	15.8	515.1195	155185496.0±32264831.5ab	136029863.3±17655081.7c	143985987.7±24016804.7bc	159663632.9±10082966.5a
	Isochlorogenic acid C	$C_{25}H_{24}O_{14}$	M-H	17.2	515.1195	42463977.5±9071142.2a	31901517.4±6438307.7c	39646469.6±11075790.6ab	35563802.7±4415221.9bc
	Feruloylcaffeoylquinic acid I	$C_{26}H_{26}O_{12}$	M-H	19.4	529.1352	2122203.8±576346.7a	1514347.6±356502.1b	1632100.7±707518.0b	1574349.9±197617.3b
	Feruloylcaffeoylquinic acid II	$C_{26}H_{26}O_{12}$	M-H	20.2	529.1352	109382.6±28070.6a	112501.6±15300.6a	111695.9±36041.6a	115312.5±11927.2a
	Feruloylcaffeoylquinic acid III	$C_{26}H_{26}O_{12}$	M-H	20.6	529.1352	476945.4±126182.0a	283536.8±69977.8b	439701.2±63596.9a	334408.0±128206.9b
	p-Coumaroyl caffeoylquinic acid I	$C_{25}H_{24}O_{11}$	M-H	18.3	499.1246	873626.5±206193.2ab	668069.1±177508.5c	766752.3±226014.4bc	916162.4±140443.6a
	p-Coumaroyl caffeoylquinic acid II	$C_{25}H_{24}O_{11}$	M-H	18.8	499.1246	228090.9±63573.9a	189454.6±47976.4b	171142.9±79525.3bc	135692.6±21637.6c
	p-Coumaroyl caffeoylquinic acid III	$C_{25}H_{24}O_{11}$	M-H	19.9	499.1246	208429.0±45165.1a	165985.6±37146.2c	183491.5±47686.5bc	195931.2±19557.2ab
	Sinapoyl caffeoylquinic acid III	$C_{27}H_{28}O_{13}$	M-H	19.9	559.1457	306034.1±155738.4a	193015.1±55419.0b	245865.7±188876.7ab	75269.5±40108.4c
	Caffeoylquinic acid-glucoside	$C_{22}H_{28}O_{14}$	M-H	3.8	515.1406	592154.2±243078.7b	1799111.2±798818.4a	1817609.1±642104.2a	2076025.9±782144.4a
	Feruloylquinic acid-glucoside	$C_{23}H_{30}O_{14}$	M-H	5.1	529.1563	56552.7±25572.1b	145428.2±53218.6a	131440.8±22038.9a	132717.6±35118.9a
	3-4-O-caffeoylquinic acid-glucoside	$C_{28}H_{38}O_{19}$	M-H	11.8	677.1723	223408.7±96846.8b	510505.1±210334.3a	634201.6±149920.0a	617873.2±203456.7a
	3-5-O-caffeoylquinic acid-glucoside	$C_{28}H_{38}O_{19}$	M-H	12.9	677.1723	103549.6±43926.9b	255449.1±89089.2a	279242.6±70515.2a	254591.4±81391.8a
	4-5-O-caffeoylquinic acid-glucoside	$C_{28}H_{38}O_{19}$	M-H	13.6	677.1723	224266.7±63606.4c	289021.9±51474.0b	325662.5±67754.5ab	335560.1±55461.2a
	p-Coumaroyl-glucoside	$C_{15}H_{18}O_8$	M-H	6.3	325.0929	173609.4±35886.7b	195194.4±23434.8a	144200.4±10761.1c	162022.3±20378.3b
	Protocatechuic acid-4-glucoside	$C_{13}H_{16}O_9$	M-H	2.5	315.0722	1648735.7±390910.5c	2450977.0±637377.9a	1959031.0±264956.4b	1837310.3±273858.1bc
	Isoeugenol	$C_{10}H_{12}O_2$	M-H	20.6	163.0765	54491.6±29041.7c	218864.8±51136.4a	58973.0±17106.9c	182608.4±46762.9b
	3-Hydroxybenzoic acid	$C_7H_6O_3$	M-H	5.2	137.0244	357674.6±101417.7bc	394129.0±60573.0ab	306759.9±106968.6c	451371.4±66234.6a
**Iridoids**	Lonijapospiroside B I	$C_{27}H_{31}NO_{12}$	M-H	16.9	560.1774	1021379.7±245918.0b	1216460.4±392404.2a	986681.6±67054.0b	1144208.0±199856.9ab

	Lonijapospiroside B II	$C_{27}H_{31}NO_{12}$	M-H	19.6	560.1774	4442736.9±1121012.4b	5327840.8±1713465.5a	4269128.6±209209.7b	4905333.4±868658.7ab
	Lonijapospiroside B III	$C_{27}H_{31}NO_{12}$	M-H	21.0	560.1774	1898241.3±480894.0a	2133777.5±681867.7a	1833711.7±88955.5a	2012315.9±354300.0a
	Secologanoside I	$C_{16}H_{22}O_{11}$	M-H	2.5	389.1089	10435034.9±4934124.5a	4089117.4±1447238.0b	9020068.2±3924727.0a	8617486.7±3570572.1a
	Secologanoside II	$C_{16}H_{22}O_{11}$	M-H	3.3	389.1089	3424037.9±1426159.0a	1535521.0±428025.2b	3318400.0±929914.8a	2846234.7±1031007.7a
	Secologanoside III	$C_{16}H_{22}O_{11}$	M-H	6.4	389.1089	40432768.3±15374205.8b	15774599.5±5907905.9c	54901078.9±22252387.7a	56396835.5±16938285.3a
	Dimethylsecologanoside I	$C_{18}H_{26}O_{11}$	M-H	11.7	417.1402	1340972.9±381737.5a	280158.9±169616.1c	1237847.5±324263.9a	516123.7±292979.6b
	Dimethylsecologanoside II	$C_{18}H_{26}O_{11}$	M-H	13.7	417.1402	67638.5±29199.1a	82455.0±71443.2a	365964.9±368977.2a	1584984.6±1108022.0b
	(E)-Aldosecologanin	$C_{34}H_{46}O_{19}$	M-H	16.9	757.2561	15515989.3±4233400.4a	7834446.5±1755409.9c	11488705.2±2731171.5b	9225348.7±1219583.6c
	(Z)-Aldosecologanin	$C_{34}H_{46}O_{19}$	M-H	18.0	757.2561	5472367.9±1808515.0a	2888470.2±597882.5c	3770516.0±1016920.7b	2713009.1±899287.8c
	Swertiamarin	$C_{16}H_{22}O_{10}$	M-H	6.9	373.1140	224040450.4±44350138.9ba	239509416.0±28159531.5b	212687700.8±9536364.5c	255358175.1±18563691.0a
	Genameside A I	$C_{17}H_{26}O_{12}$	M-H	3.5	421.1352	607857.6±343702.1a	200642.1±43501.7b	532187.3±329404.6a	559005.2±264256.4a
	Genameside A II	$C_{17}H_{26}O_{12}$	M-H	4.1	421.1352	3261355.5±1121829.9b	4469736.2±1664360.4a	1759652.3±791180.2c	3331321.0±782269.0b
	Genameside A III	$C_{17}H_{26}O_{12}$	M-H	5.3	421.1352	272231.7±85573.1b	345554.0±193184.0b	553167.0±306703.0a	309411.7±93333.0b
	8-Epi-loganic acid	$C_{16}H_{24}O_{10}$	M-H	4.4	375.1297	2199088.1±740228.4a	1354648.1±365226.0b	2278674.3±453552.4a	2197012.9±536311.2a
	7-Epi-loganic acid	$C_{16}H_{24}O_{10}$	M-H	5.3	375.1297	8878273.3±3120375.6b	12000547.4±7494830.3b	20147645.5±12411361.8a	10528978.3±3616536.2b
	Arbutoside I	$C_{28}H_{42}O_{20}$	M-H	7.6	697.2197	6359243.0±1852186.5b	1821597.4±634096.7d	7976027.2±1029716.9a	4108307.3±935763.4c
	Arbutoside II	$C_{28}H_{42}O_{20}$	M-H	8.1	697.2197	3701908.0±1051711.3b	1394449.8±490414.4d	4565981.3±483191.9c	3004537.5±645839.8a
	Demethyl-strychoside A	$C_{32}H_{42}O_{19}$	M-H	12.5	729.2248	11794104.7±3968463.9b	10368824.3±3903433.4b	9905529.2±2246089.0b	14747989.7±2843077.1a
	7-O-Methyl morroniside	$C_{18}H_{28}O_{11}$	M-H	10.0	419.1559	51687.4±25598.9b	38582.4±13847.3b	52859.4±14667.8b	76046.2±24071.3a
	Strychoside A I	$C_{33}H_{44}O_{19}$	M-H	14.3	743.2404	7949562.7±2401249.2b	5587959.7±1623379.5b	6389780.1±1532039.5b	5445103.3±559422.2a
	Strychoside A II	$C_{33}H_{44}O_{19}$	M-H	14.8	743.2404	9090731.2±2358337.5b	6495791.4±1650262.7b	6818964.5±1686726.1b	6033882.1±726197.3a
**Fatty acids**	FA 16:1	$C_{16}H_{30}O_2$	M-H	34.1	253.2173	204097.3±64989.9b	224915.7±38471.6b	403363.9±88013.7a	416102.4±64224.8a
	FA 18:0	$C_{18}H_{36}O_2$	M-H	37.0	283.2643	18905979.5±5498313.0c	17568310.9±3075309.6c	26764310.5±13864058.9b	32146155.7±6142203.4a
	FA 18:1	$C_{18}H_{34}O_2$	M-H	35.6	281.2486	2928644.4±1116521.6b	1646556.6±390217.9c	4784037.1±2062426.2a	3538350.3±785320.3b
	FA 18:2	$C_{18}H_{32}O_2$	M-H	34.4	279.2330	8938502.1±3301480.7a	3204598.5±1146606.5c	9605681.3±5017431.1a	6550958.7±2072988.8b
	FA 18:3	$C_{18}H_{30}O_2$	M-H	33.3	277.2173	6502823.0±2531720.5ab	3080018.7±1457876.4c	7934466.8±3594453.9a	5725513.7±1195815.2b
	FA 20:0	$C_{20}H_{40}O_2$	M-H	31.8	311.2956	52790.6±31648.0b	160211.9±102397.2a	155936.5±56412.1a	223189.4±148005.7a
**Others**	Pinoresinol-*4-O*-glucoside	$C_{26}H_{32}O_{11}$	M-H	15.3	519.1872	260416.1±63526.4c	389630.0±88441.5a	319710.9±56739.2b	373196.1±65715.4a

	Abs*cis*ic acid	$C_{15}H_{20}O_4$	M-H	20.1	263.1289	284091.3±81710.8c	505675.6±220144.7ab	450228.8±98113.2b	619251.2±256162.6a
	2-Isopropylmalic acid	$C_7H_{12}O_5$	M-H	5.1	175.0612	136711.6±28259.2c	207270.2±91974.3ab	184429.0±39399.3bc	239642.6±87017.3a
	Galactonic acid	$C_6H_{12}O_7$	M-H	0.8	195.0510	2375850.0±1293593.3b	2118990.7±435061.1b	3606987.6±692263.4a	3408554.5±1051693.0a
	Pantothenic acid	$C_9H_{17}NO_5$	M-H	2.7	218.1034	316802.8±72143.6b	389062.9±77821.7a	378683.7±41357.9a	406596.2±50947.8a
	Dichlorogelignate	$C_{32}H_{34}O_{18}$	M-H	5.2	705.1672	2466734.6±1975567.2bc	1667486.3±237156.5c	3682003.4±1212468.4a	2780377.1±770675.2ab
	Glucose	$C_6H_{12}O_6$	M-H	0.7	179.0561	10786249.9±3098108.6c	13827986.3±2121882.6a	12291766.3±624295.7b	11654374.5±1664245.9bc
	Citric acid	$C_6H_8O_7$	M-H	6.2	191.0197	96181.9±30621.8b	112755.9±13319.6a	117878.2±15451.6a	116387.6±19310.8a
	Malic acid	$C_4H_6O_5$	M-H	0.9	133.0143	22550967.7±5542857.0ab	21738397.1±5844481.5b	25287128.9±6116719.3a	20594479.9±3475850.2b
	Fumaric acid	$C_4H_4O_4$	M-H	0.9	115.0037	6914585.5±1661637.2ab	6550487.2±1868883.0ab	7591294.4±1814836.4a	6141181.1±924613.0b
	Quinic acid	$C_7H_{12}O_6$	M-H	0.8	191.0561	143522781.3±27311344.5a	136129223.5±5102890.5bc	140486629.3±10117131.7ab	133925856.0±9077983.3c
**Flavonoids**	Diosmetin-Oglucoside I	$C_{22}H_{22}O_{11}$	M+H	17.2	463.1240	103672.3±22402.6b	172775.4±48368.0b	402986.6±317152.2a	169038.8±97129.9b
	Diosmetin-Oglucoside II	$C_{22}H_{22}O_{11}$	M+H	17.7	463.1242	15253.5±3531.3b	28313.4±10211.4a	30787.6±10555.6b	20677.3±5046.1a
	Corymbosin	$C_{19}H_{18}O_7$	M+H	29.2	359.1124	195773.0±50072.3b	138361.8±34506.6b	510152.2±419542.9a	204622.9±72625.8b
	Flavoyadorinin B	$C_{23}H_{24}O_{11}$	M+H	23.3	477.1374	222883.0±57747.2a	147930.0±46036.0b	170769.3±42809.4b	93254.3±31289.2c
**Phenolic acids**	Caffeic acid	$C_9H_8O_4$	M+H	5.8	181.0480	20626.0±5036.7c	34583.2±14858.6bc	43279.3±16830.4b	85369.9±32975.0a
	Ferulic acid	$C_{10}H_{10}O_4$	M+H	6.9	195.0640	7441328.5±1627799.9b	8194260.7±1230477.4ab	8011311.1±1008544.6b	8832303.4±1110195.4a
**Iridoids**	Loganic acid-*O*-pentoside	$C_{21}H_{32}O_{14}$	M+H	7.4	509.1850	411711.2±105859.9b	327370.2±180802.5b	373326.3±154404.0b	573851.9±177496.8a
	Secoxyloganic I	$C_{17}H_{24}O_{11}$	M+H	5.5	405.1372	1551283.5±480399.7a	1537145.6±409128.2a	995012.7±314810.4b	1440052.2±354956.1a
	Secoxyloganic II	$C_{17}H_{24}O_{11}$	M+H	7.1	405.1378	796584.1±274352.4a	855109.3±232433.4a	472077.7±197834.6b	751841.7±167235.3a
	Secoxyloganic III	$C_{17}H_{24}O_{11}$	M+H	10.5	405.1390	2243460.2±691140.7b	2609382.6±1056709.1b	2413976.2±476537.0b	4316875.5±1857285.4a
	Loganic I	$C_{17}H_{26}O_{10}$	M+H	9.0	391.1595	44004.9±11796.9a	44088.2±11288.6a	44192.2±17099.2a	39958.4±6166.2a
	Loganic II	$C_{17}H_{26}O_{10}$	M+H	10.2	391.1595	1611640.5±744294.1b	2865103.7±835099.5a	890136.7±176170.2c	1396877.6±613505.5b
	5a-Carboxystrictosidine I	$C_{28}H_{34}N_2O_{11}$	M+H	12.9	575.2234	99112.5±37930.6c	215081.8±42538.2b	131291.9±34233.3c	320935.9±114014.3a
	5a-Carboxystrictosidine II	$C_{28}H_{34}N_2O_{11}$	M+H	14.6	575.2234	2317090.6±552606.2bc	2022015.3±289242.6c	3000850.6±491276.8a	2545809.3±589755.6b
	5a-Carboxystrictosidine III	$C_{28}H_{34}N_2O_{11}$	M+H	15.7	575.2231	63994.0±29687.7c	169717.2±29922.1b	66668.6±17964.3c	216961.5±81468.2a
	5a-Carboxystrictosidine IV	$C_{28}H_{34}N_2O_{11}$	M+H	17.5	575.2233	1511767.1±376400.9bc	1374600.7±201115.9c	1930778.4±275225.7a	1639471.5±389540.6b
	Hydro-dimethyl lonijaposide C	$C_{26}H_{35}NO_{12}$	M+H	8.4	554.2222	778922.6±217749.9b	569937.8±215895.0c	760174.5±145266.1b	1118612.4±324061.3a
	Vogeloside I	$C_{17}H_{24}O_{10}$	M+H	11.2	389.1443	16068707.1±5500777.1b	20758884.3±2617605.4a	12709381.5±1302122.7c	19723930.8±3126852.8a

	Vogeloside II	$C_{17}H_{24}O_{10}$	M+H	10.4	389.1436	807166.9±331949.8bc	690043.5±145874.1c	900516.4±180040.9ab	1061446.7±290196.4a
	7-O-Ethylsweroside	$C_{18}H_{26}O_{10}$	M+H	15.2	403.1599	347856.7±132375.6b	320945.4±66443.9b	290429.5±26683.7b	441132.1±108643.4a
	Lonijaposide T	$C_{28}H_{35}NO_{13}$	M+H	10.8	594.2174	7469332.4±2285193.2a	1315668.6±505921.2c	6209803.1±1774854.1b	1925755.9±1080301.4a
	Lonijaposide B	$C_{25}H_{31}NO_{12}$	M+H	12.2	538.2275	17725442.0±3832322.4a	11810729.9±5943829.3b	11291527.8±4208640.8b	13332889.8±3757434.9b
	Dimethyl lonijaposide C	$C_{26}H_{33}NO_{12}$	M+H	9.2	552.2062	6649694.4±2804563.0a	1469953.9±409108.8b	5624222.5±1687267.2a	2405879.5±933834.2b
	Sweroside	$C_{16}H_{22}O_{9}$	M+H	8.7	359.1329	4645954.4±1528888.2a	1473125.7±248718.8c	4016517.3±960127.0a	2155090.1±475660.5b
**Amino acids**	Serine	$C_{3}H_{7}NO_{3}$	M+H	0.8	106.0500	284990.2±66168.9c	400167.4±47094.2b	376067.8±71849.5b	546736.4±106243.8a
	Phenylalanine	$C_{9}H_{11}NO_{2}$	M+H	2.1	166.0861	57866.1±13350.8b	72195.0±17836.6a	60848.8±10837.5b	67554.9±19092.9ab
	Tyrosine	$C_{9}H_{11}NO_{3}$	M+H	0.9	182.0811	270003.4±56378.9b	323378.2±69530.9a	275581.4±25184.7b	352739.0±77586.3a
	Valine	$C_{5}H_{11}NO_{2}$	M+H	0.9	118.0864	290149.6±61753.5c	405868.1±86252.5a	340079.4±36919.6b	415409.8±67486.7a
	Leucine	$C_{6}H_{13}NO_{2}$	M+H	1.3	132.1021	71228.7±16522.2c	87465.6±23296.8b	94916.7±18117.6b	118404.2±24149.8a
	Proline	$C_{5}H_{9}NO_{2}$	M+H	0.8	116.0707	1785518.1±395902.6c	2035435.4±297412.2b	2069941.1±244592.4b	2568892.2±343937.8a
	Tryptophan	$C_{11}H_{12}N_{2}O_{2}$	M+H	4.0	205.0965	141444.0±52047.7d	402211.9±75287.7a	207362.7±62775.7c	304215.3±62901.6b
	Adenosine monophosphate	$C_{10}H_{14}N_{5}O_{7}P$	M+H	1.0	348.0705	227845.7±72109.3b	266578.2±30385.7a	188851.7±55197.8c	204195.5±28286.5bc
	Guanosine	$C_{10}H_{13}N_{5}O_{5}$	M+H	1.3	284.0994	503079.2±169890.5a	540240.4±214664.1a	492243.6±126912.7a	215423.3±215181.1b
	Guanine	$C_{5}H_{5}N_{5}O$	M+H	1.3	152.0569	980117.6±331180.8a	1040461.6±405863.2a	938146.7±236102.6a	413515.5±416086.2b
	Adenine	$C_{5}H_{5}N_{5}$	M+H	1.2	136.0619	389339.5±88983.4a	384211.6±107963.2a	386585.9±93641.0a	170860.8±112591.6b
	Adenosine	$C_{10}H_{13}N_{5}O_{4}$	M+H	1.2	268.1043	1896455.3±429116.4a	1859344.6±485099.2a	1912088.3±430050.4a	851732.8±576822.1b
	5'-Methylthioadenosine	$C_{11}H_{15}N_{5}O_{3}S$	M+H	4.4	298.0959	87357.3±57339.1b	202309.9±84818.1a	59256.5±18296.4b	100790.4±37294.3b
**Lipids**	Lyso PC 18:3 sn-2	$C_{26}H_{48}NO_{7}P$	M+H	28.6	518.3239	2155204.8±766270.0a	1627595.1±435779.3b	2206747.3±594554.7a	1304438.9±691644.3b
	Lyso PE 16:0 sn-1	$C_{21}H_{44}NO_{7}P$	M+H	29.1	454.2927	27214.3±6662.0c	82085.8±26243.7a	30739.8±4483.9c	66560.3±27109.8b
	Lyso PE 16:0 sn-2	$C_{21}H_{44}NO_{7}P$	M+H	29.4	454.2927	428590.3±116072.6b	1243703.9±387313.2a	498165.5±71471.7b	1077451.0±434310.3a
	Lyso PE 18:0	$C_{23}H_{48}NO_{7}P$	M+H	31.4	482.3241	69927.9±26787.6b	185801.6±56156.6a	83604.3±17345.4b	208680.2±74969.3a
	Lyso PE 18:1	$C_{23}H_{46}NO_{7}P$	M+H	29.9	480.3086	56123.2±12583.9bc	100823.0±29016.6a	52671.7±12190.2c	70825.1±23200.6b
	Lyso PE 18:2sn-1	$C_{23}H_{44}NO_{7}P$	M+H	28.6	478.2929	49606.1±12252.0bc	89358.1±27723.2a	42474.1±11813.5c	57171.0±16761.7b
	Lyso PE 18:2sn-2	$C_{23}H_{44}NO_{7}P$	M+H	28.8	478.2929	416351.3±122834.2b	527303.7±199200.8a	400665.2±99756.4b	443330.6±158409.1ab
	Lyso PE 18:3sn-1	$C_{23}H_{42}NO_{7}P$	M+H	27.7	476.2773	27488.8±8044.8b	48961.2±12789.3a	25593.1±7703.0b	25716.2±11276.0b
	Lyso PE 18:3sn-2	$C_{23}H_{42}NO_{7}P$	M+H	28.0	476.2773	277298.1±88510.6a	281431.4±104079.8a	246685.0±78475.7a	227617.1±117061.5a

Note: Values with different letters in a row indicated significant differences using analysis of variance (ANOVA) ($p < 0.05$).

Table S6. The details of HS-GC-IMS, HS-SPME-GC-MS, and UPLC-Q-TOF-MS.

Method	Item	Details
HS-GC-IMS	HS-GC-IMS system	FlavourSpec® flavor analyzer (G.A.S. Dortmund, Germany)
	Capillary column	MXT-WAX capillary column (30 m × 0.53 mm, 1.0 µm, Restek, USA)
	Carrier gas	Nitrogen (99.999% purity)
	Programmed flow rate	0min: 2.0 mL/min;
		0-5min: linear increase to 10.0 mL/min;
		5-35min: linear increase to 100.0 mL/min.
HS-SPME-GC-MS	SPME Fiber	50/30 µm DVB/CAR/PDMS fiber (Supelco, Bellefonte PA, USA)
	GC-MS System	GC-MS-QP 2020NX (Shimadzu Corporation, Tokyo, Japan)
	Capillary Column	DB-5 MS capillary column (30 m × 0.25 mm × 0.25 µm, Agilent, USA)
	Carrier Gas	Helium (99.999% purity)
	MS Parameters	Electron impact ionization mode;
		electron energy: 70 eV;
		mass scan range: 29–500 m/z;
		ion source temperature: 230 °C.
	Column Oven Program	0-2min: held at 40 °C for 2min;
		2-30min: ramp to 180 °C at 5 °C/min;
		30-40min: ramp to 280 °C at 10 °C/min;
		40-50min: held at 280°C for 10min.
UPLC-Q-TOF-MS	UHPLC System	H-Class (Waters, Milford, MA, USA)
	Q-TOF Mass Spectrometer	Impact II with ESI interface (Bruker, Germany)
	Column Specification	Agilent ZORBAX SB C18 column (2.1 × 100 mm, 1.8 µm, Agilent, Palo Alto, CA, USA)

Elution Gradient	Mobile phase B:<br>0-20min: increased linearly from 5% to 25%;<br>20-35min: increased linearly from 25%→100%<br>35-40min: held at 100% for 5 min;<br>40-40.1min: decreased linearly from100%→5%;<br>40.1-45min: held at 5% for 5 min.
MS Parameters	Nebulizer Pressure: 2.0 bar;<br>Drying Gas Temperature: 200 °C;<br>Collision Radio Frequency: 750 Vpp;<br>Dry Gas Flow Rate: 8.0 L/min;<br>Capillary Voltage: 3000 V (negative mode) and 3500 V (positive mode);<br>Transfer Time: 80 µs;<br>Prepulse Storage Time: 8 µs.
